# A computational deep learning investigation of animacy perception in the human brain

**DOI:** 10.1038/s42003-024-07415-8

**Published:** 2024-12-31

**Authors:** Stefanie Duyck, Andrea I. Costantino, Stefania Bracci, Hans Op de Beeck

**Affiliations:** 1 Brain and Cognition, Faculty of Psychology and Educational Sciences, KU Leuven, Leuven, Belgium; 2 Center for Mind/Brain Sciences (CIMeC), University of Trento, Trento, Italy

**Keywords:** Perception, Computational neuroscience

## Abstract

The functional organization of the human object vision pathway distinguishes between animate and inanimate objects. To understand animacy perception, we explore the case of zoomorphic objects resembling animals. While the perception of these objects as animal-like seems obvious to humans, such “Animal bias” is a striking discrepancy between the human brain and deep neural networks (DNNs). We computationally investigated the potential origins of this bias. We successfully induced this bias in DNNs trained explicitly with zoomorphic objects. Alternative training schedules failed to cause an Animal bias. We considered the superordinate distinction between animate and inanimate classes, the sensitivity for faces and bodies, the bias for shape over texture, the role of ecologically valid categories, recurrent connections, and language-informed visual processing. These findings provide computational support that the Animal bias for zoomorphic objects is a unique property of human perception yet can be explained by human learning history.

## Abstract (web summary)

Explicit training with zoo-morphic objects—as opposed to other training regimes—uniquely induces a human-like “Animal bias" in DNNs, suggesting that this bias in humans can be explained by humans’ learning history.

## Introduction

The distinction between animals and other object categories dominates functional selectivity in the human visual cortex. In occipitotemporal cortex (VTC) there are distinct neural representations of categories such as faces^1,2^, bodies^3,4^, and animals^5,6^. At the broader level, studies have found a hierarchically organized animacy continuum in VTC^7–12^, which has been related to perceptual and/or conceptual properties of animals^9,10,13–15^. A particularly interesting test case concerns zoomorphic objects: objects made to look like animals, such as a cuddly toy or a butterfly-shaped rattle. This practice might be as old as human culture itself, judging from archeological research that has revealed plenty of prehistoric artefacts with animal characteristics, e.g., pottery vessels and jewelry. In addition, the human mind does not need many cues to interpret even simple visual shapes as animate, such as when two triangles chase a square^16–19^. Previous research has suggested various benefits and origins of this animacy perception^20,21^.

Thanks to their inherent object-animal ambiguity, zoomorphic objects have turned out to be a perfect condition to reveal fundamental and unique properties of human animacy perception, reminiscent of how the study of ambiguous figures and illusions has taken up a central place in sensory and cognitive psychology. Through recent research we moved closer to a neuroscientific understanding of the tendency of human observers to interpret zoomorphic objects as animate^13^. Representational similarity analyses showed that VTC represents zoomorphic objects more like animals than like regular objects, which we here refer to as an “Animal bias”. The effect is reminiscent of other findings with face pareidolia^22^, the tendency to see faces in objects: VTC tends to represent objects that trigger face pareidolia a bit more like faces than other objects. However, the bias was much more prominent with zoomorphic objects^13^: Zoomorphic objects were represented as animals, not somewhere in between animals and regular objects.

Despite this progress, these neuroscientific investigations also exposed the extent to which we lack a computational understanding of why the human visual system is so prone to this Animal bias. Many recent studies have suggested that DNNs trained in image classification provide a useful computational model of human vision^13,23–28^. More specifically, the later layers (i.e., fully connected or deep layers) of these networks typically show a similar functional complexity^29,30^ and representational similarity structure^31^ as observed in VTC, including a rudimentary animacy continuum representation^10,25,27^. Nevertheless, there is a striking discrepancy between human and DNN vision^32,33^, particularly when it comes to zoomorphic objects. DNNs show the exact opposite bias from human visual cortex, and represent zoomorphic objects as inanimate, even as inanimate as regular objects, a so-called Object bias^13^. Because of this failure, we do not understand what induces the Animal bias in human perception and neural responses. Is the human Animal bias a consequence of prior exposure to or learned importance of zoomorphic objects, in development or at an evolutionary time scale? Might the Animal bias be a side effect of one of the many other ways in which visual information processing in the human brain differs from artificial neural networks, such as a different weight given to shape and texture features^34^? Or is the Animal bias due to the known differences in architecture between artificial and biological brains, including the lack of recurrent processing in the most common artificial networks?

Finding an answer to these questions is notoriously difficult because of the complexity of human object representations^35^ and the challenge to experimentally dissociate all the possibly relevant properties of these representations. DNNs provide unique opportunities to investigate under which conditions this representational phenomenon occurs and how it possibly relates to other representational properties. This is a very different mindset compared to the large number of prior studies that show that DNN models are often able to predict human object representations at the neural and behavioral level for generic stimulus sets^27,36–38^. This focus upon prediction has been criticized, and it was suggested that studies should focus upon specific psychological phenomena instead^39^. Here we combine the best of both worlds: We single out one particularly revealing and seemingly unique property of human visual information processing, and we take advantage of the high capacity of deep learning models to investigate the role of multiple characteristics of object representations.

Here we apply this approach to understand what it takes to construct a computational model that represents zoomorphic objects in a similar manner as the human visual system, with an Animal bias rather than an Object bias. We test several hypotheses. First, we investigate whether it is possible to induce an Animal bias in DNNs by explicitly training DNNs to categorize zoomorphic objects as animals, contrary to their initial Object bias. This effort was successful. This success suggests a first hypothesis for why the adult human brain might show a strong Animal bias: because of the frequent encounter of zoomorphic objects meant to represent animals.

Next, we test other hypotheses rooted in well-documented properties of the human visual system that might also explain an Animal bias. In comparison to DNNs, human vision has been shown to be particularly sensitive to the superordinate distinction between animals and other objects^7–10^, to categories like faces and bodies^1–4^, to global shape^40^, and to the natural statistics in the environment^41^. In addition, human vision involves recurrent processing^42–47^ and seems to be further tuned by the development of language^48^. We tested computational models constructed to include these properties. None of them displayed an Animal bias in DNNs, nor captured brain representations well. Through these computational tests we narrow down the possible causes of the Animal bias to the relevance of zoomorphic objects during past training of the human visual system. These findings reveal the potential of deep learning models to probe the multiverse of mechanisms that might underlie important properties of human object perception.

## Results

### Fine-tuning can shift the representational space for zoomorphic objects in pretrained DNNs

**Fig. 1:**
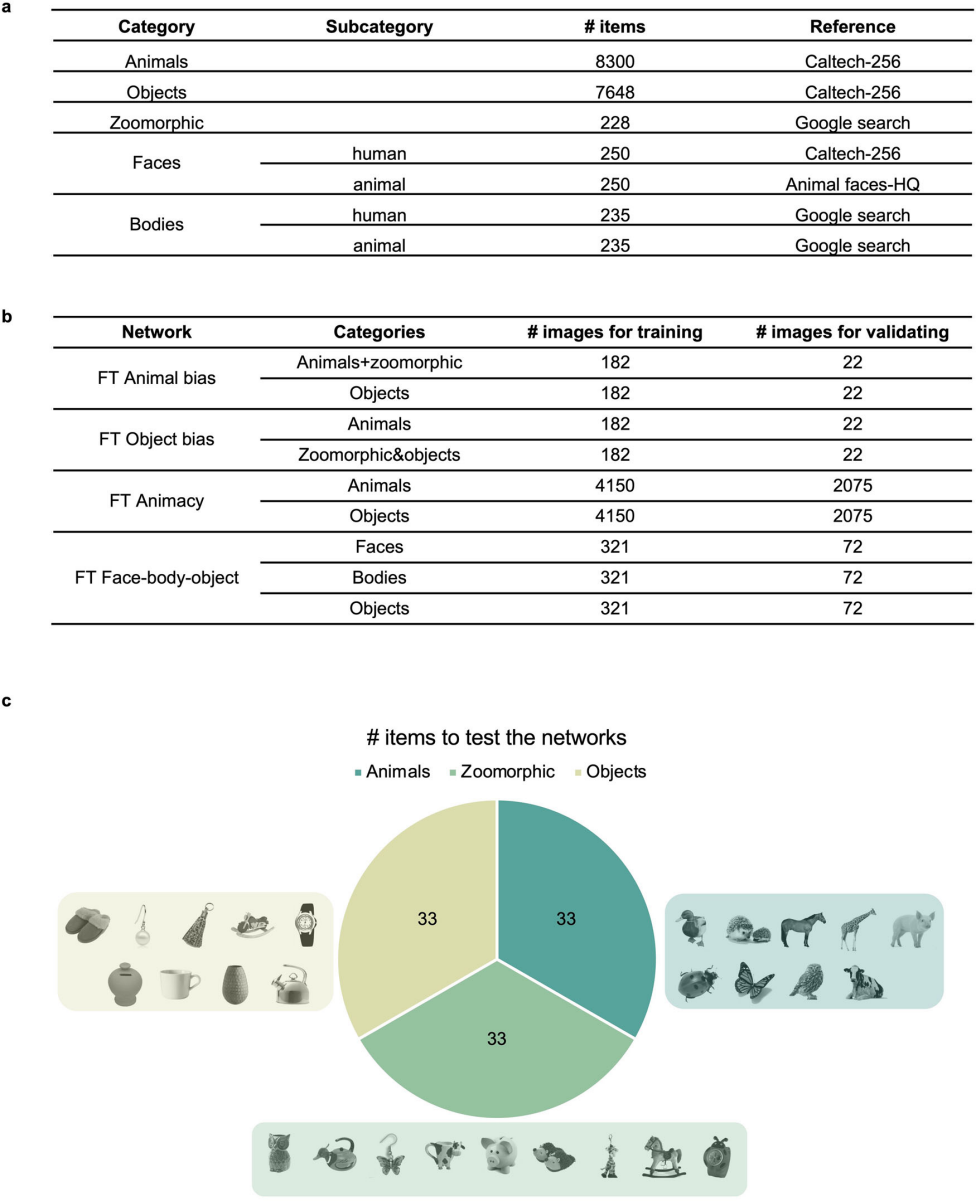
Overview of the stimuli. **a** Info on the whereabouts and numbers of all image categories. **b** Overview of categories in each type of training regime and the number of images used for training and validating. **c** An overview of the number of images in the test phase, used to construct the Representational Similarity Matrices. 33 images in each category, of which the 9 Bracci subset images are displayed for each category (stimuli images adapted from ref. ^13^).

We first investigated the extent to which the clustering of zoomorphic objects with either animals (animal bias) or objects (object bias) can be influenced by directly training a neural network using zoomorphic objects. To shift the representational space for zoomorphic objects, pretrained AlexNet networks (PT ImageNet) were fine-tuned (FT) over the last two fully connected layers (i.e., FC7 and FC8). A dataset with three separate categories (i.e., animals, zoomorphic objects and objects) was split for training, validating and testing the networks (Fig. 1a). Depending on the type of training (Fig. 1b), two out of three categories were joined together. The FT Animal bias DNN was trained to classify images either as animals plus zoomorphic or as objects. Conversely, FT Object bias DNN, was trained on animals versus zoomorphic plus objects. The test set contained images from earlier work^13^ (Fig. 1c) and new ones (for a total of 99 test images, 33 per category).

**Fig. 2:**
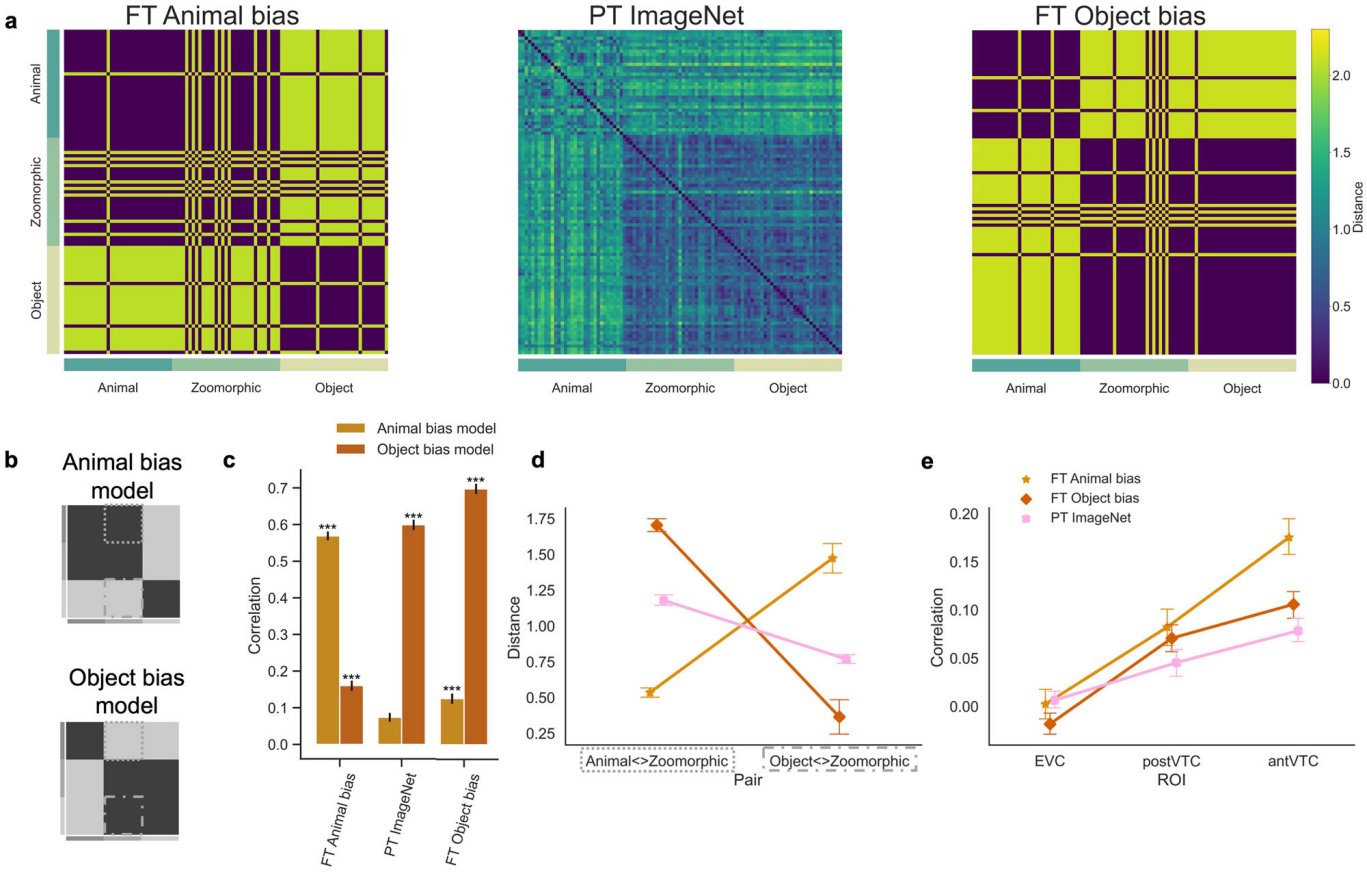
Overview of the explicit Animal bias training results. **a** The Representational Similarity Matrices in FC8 of three neural networks included in the analyses are displayed. FT = fine-tuned AlexNet, PT = pretrained AlexNet. For FC8 in these networks the RDMs are binary because there are only 2 output units. **b** Graphical illustration of the pre-defined conceptual Animal and Object bias model. **c** The graph represents the correlation for each neural network with each bias model. Significant values (i.e., ***p < 0.0001, **p < 0.001, *p < 0.01), were computed with permutation tests (10,000 randomizations), and error bars indicate standard error computed via bootstrapping. **d** Mean distance score of each image in the contrast Animal < > Zoomorphic and Object < > Zoomorphic (see dotted outlines in panel b) and this for each neural network. For violin plots showing the full distributions, please see Supplementary Fig. 4. **e** Graph with the correlations between the individual neural data in the three regions of interest (i.e., EVC, posterior and anterior VTC) and the neural network data resulting from the different training regimes. All error bars represent the standard error.

Analyses on DNNs data were performed on the entire test set, whereas analyses investigating the correlations with the neural data were performed on the subset derived from earlier work. All results are based upon the normalized distance ((1−*R*_Pearson_)/*meanRDM*) between the output representations of all image pairs, resulting in representational dissimilarity matrices (Fig. 2a).

The RDM matrices showed a strong effect of the fine-tuning training protocol in terms of correlations with pre-specified conceptual representational structures (see Fig. 2b). These aligned either with an Object bias (Object bias model) or with a human-like Animal bias (Animal bias model). More specifically, as shown in Fig. 2c, the object space derived from the FT Animal bias DNNs exhibited a strong correlation with the Animal bias model (*r* = 0.57), which was significantly higher compared to the Object bias model (*r* = 0.16; *p* < 0.001). Conversely, the FT Object bias DNN showed a strong correlation with the Object bias model (*r* = 0.70) compared to the Animal bias model (*r* = 0.13; *p* < 0.001). The representational space from the baseline PT ImageNet network also exhibited a stronger correlation with the Object bias model (*r* = 0.60) relative to the Animal bias model (*r* = 0.07; *p* < 0.001). We limited our reporting in this Results section to one network architecture (AlexNet) and its last network layer (FC8 in AlexNet). However, similar trends, albeit with smaller effect sizes, were present in layer FC7 (see Supplementary Fig. 2). These findings were further supported by simulations using another frequently employed architecture (VGG; see Supplementary Fig. 1).

In addition to correlations with the independent models, that include all non-diagonal cells in the RDM, we specifically investigated (Fig. 2d) the distance between animal and zoomorphic objects, and between regular and zoomorphic objects, both averaged across all pair-wise image comparisons (see dotted outlines in Fig. 2b). The smaller the distance, the more similar the representations of both categories are. An animal bias would manifest itself by a smaller distance in Animal < > Zoomorphic than in Zoomorphic < > Object, and visually by a positive slope in Fig. 2d. A factorial repeated-measures ANOVA on the dissimilarity scores with factors Distances (Animal < > Zoomorphic; Object < > Zoomorphic) x Network (PT ImageNet; FT Object bias; FT Animal bias) revealed a significant interaction effect (*F*(2, 64) = 94.88, *p* < 0.001). Post-hoc analyses further investigated the degree to which the different networks learnt to consider zoomorphic objects more like animals or objects. Relative to the pretrained PT ImageNet DNN, the fine tuning in the FT Animal bias DNN moved the zoomorphic objects closer to the animals (*M* = 0.53, *t*(32) = 20.07, *pcorr* < 0.001, *BF*_10_ = 4.3 × 10^16^; See Supplementary section 4 for the full Bayes Factors matrix.) and further away from objects (*M* = 1.47, *t*(32) = −5.15, *pcorr* < 0.001, *BF*_10_ = 1.1 × 10^4^). The reverse is observed for the FT Object bias DNN, with zoomorphic farther from animals (*M* = 1.70, *t*(32) = −6.06, *pcorr* < 0.001, *BF*_10_ = 1.3 × 10^7^) and closer to objects (*M* = 0.37, *t*(32) = 4.26, *pcorr* < 0.001, *BF*_10_ = 13) compared to PT ImageNet. In sum, the fine-tuned networks shifted towards the bias they were trained in, with a particularly large effect size for the Animal bias training.

### Animal bias DNN captures brain representations better than Object bias DNNs

The earlier finding^13^ of a strong Animal bias in anterior VTC, makes us predict that the FT Animal bias DNN should correlate well with the representational similarity in ant VTC. The individual RDMs (*n* = 16) of each region of interest (early visual cortex (EVC), anterior and posterior VTC) were correlated with the DNN RDMs (Fig. 2e). In VTC, in contrast to EVC, all DNNs showed a positive correlation (one-sided one-sample t-test, all *p* ≤ 0.003) with the neural data in anterior and posterior VTC. This result converges with previous results showing that fully connected layers in DNNs better predict brain representations in high-level visual areas relative to low-level visual cortex^49^. Importantly, we observed by means of a repeated-measures ANOVA, a significant interaction between ROI (EVC; ant VTC; post VTC) x Network (FT Animal bias; PT ImageNet; FT Object bias) (*F*(2, 60) = 7.93, *p* = 0.002). Post-hoc analyses provided insight into representations learned by the FT Animal bias DNN, such that these were significantly more correlated with representations in ant VTC (*r* = 0.18), relative to representations learned by FT Object bias (*r* = 0.11, *t*(15) = 3.86, *pcorr* = 0.007) and the standard PT ImageNet DNN (*r* = 0.08, *t*(15) = 5.34, *pcorr* < 0.001). This difference was not observed in post-VTC (Object bias: *r* = 0.07, *t*(15) = 0.89, *pcorr* = 0.43; PT ImageNet: *r* = 0.05, *t*(15) = 2.47, *pcorr* = 0.08). Together these results show that a DNN trained to classify zoomorphic objects like animals can capture the human anterior VTC representations much better than DNNs without such training.

Overall, the findings obtained after training a network to classify zoomorphic objects as animals, showed that DNNs can show an Animal bias and that the resulting representational structure mimics brain representations to a greater extent relative to benchmark DNNs. Clearly, both an Animal bias and an Object bias can emerge in a high-capacity information processing system. The main question emerging from the human literature is: Why does the human visual system show an Animal bias? The DNN findings provide already one obvious hypothesis: As we mimicked in the FT Animal Bias training, the human Animal bias might also result from exposure to zoomorphic objects and being rewarded during development for considering such objects as animal-like. However, the failure of benchmark DNNs to show this Animal bias might also have resulted from other obvious and previously described differences in the visual input and tasks that humans and artificial systems are exposed to. These differences are more generic than what happens with zoomorphic objects, but they might indirectly cause a particular processing of such objects. In the following sections, we test several proposals. In each case, we compare an additional DNN trained to test the hypothesis at hand with the baseline PT ImageNet DNN and the FT Animal bias DNN. As such, all ANOVA analyses with the factor ‘Network’ consist of three levels: FT Animal bias DNN, PT ImageNet DNN and the newly added DNN.

### Hypothesis one: Animal bias because of explicit training in categories such as animacy or faces and bodies

The human visual system is particularly sensitive for the distinction between inanimate and animate objects, the latter represented as a continuum^7–10^, and shows regions with a very strong selectivity for faces and bodies^3^. However, these two types of selectivity are only partially captured by benchmark DNNs, possibly due to limitations in their training history. First, whereas the animacy continuum is present in DNNs, the categorical distinction seems less pronounced than in the human brain^25^. DNNs are trained on very specific labels (e.g., specific species of dogs and birds) and not so much on super-ordinate categories (e.g., animate). Remediating this can in some training regimes improve the fit between DNN representations and responses in visual cortex^50^. Fine-tuning the PT ImageNet in the super-ordinate animate-inanimate distinction (FT Animacy), without the inclusion of zoomorphic stimuli, might give rise to more Animal bias and convergence with VTC for zoomorphic objects.

Second, whereas face and body selectivity are very prominent in the human visual system, these two categories are not included as explicit classes in the typical ImageNet training protocol. Given that face and body selectivity is an important determinant of animacy representations^10^, it might also underlie the Animal bias. We tested whether fine-tuning a DNN with human and animal faces and bodies, in addition to objects, might induce an Animal bias (FT Face-body-object).

**Fig. 3:**
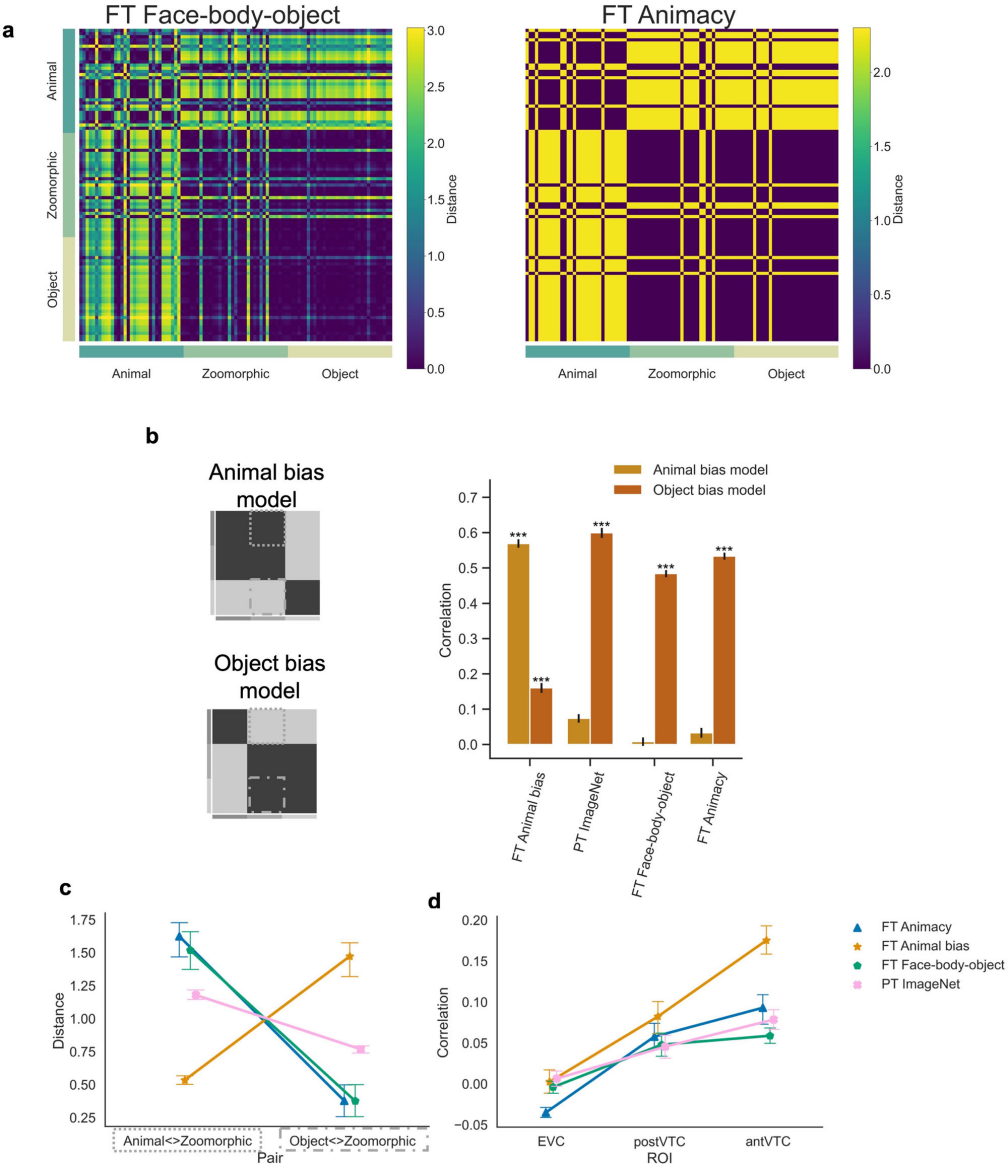
Alternative hypothesis one: sensitivity for specific categories. **a** The Representational Similarity Matrices of two alternative networks included in the analyses are displayed. FT = fine-tuned AlexNet, PT = pretrained AlexNet. **b** Graphical illustration of the pre-defined conceptual Animal and Object bias model. The graph represents the correlation for each neural network with each bias model. Significant values (i.e., ***p < 0.0001, **p < 0.001, *p < 0.01), were computed with permutation tests (10,000 randomizations), and error bars indicate standard error computed via bootstrapping. **c** Mean distance score of each image in the contrast Animal < > Zoomorphic and Object < > Zoomorphic and this for each neural network. For violin plots showing the full distributions, please see Supplementary Fig. 4. **d** Graph with the individual correlations between the neural data in the three regions of interest (i.e., EVC, posterior- and anterior VTC) and the neural network data resulting from the different training regimes. All error bars represent the standard error. Note that in this figure and all following figures, the data shown for FT Animal Bias and FT ImageNet are the same as in Fig. 2. We repeat these models to ease comparison with the models added to test the specific hypotheses in later figures.

Figure 3 displays the results for FT Animacy and FT Face-body-object, together with the DNNs, PT ImageNet and FT Animal bias. The RDMs from the alternative networks are shown in Fig. 3a, where we can already observe that the representational structure in the two new DNNs tends to align with PT ImageNet and that FT Animal bias stands apart as the odd-one-out. Quantitative analyses revealed strong positive correlations for both alternative DNNs with the independent Object bias model (Fig. 3b; FT Animacy DNN: *r* = 0.53; FT Face-body-object DNN: *r* = 0.48) relative to the Animal bias model (all *p* < 0.001) which did not differ from zero (FT Animacy DNN: *r* = 0.03, *p* = 0.10; FT Face-body-object DNN: *r* = 0.008, *p* = 0.34).

Next, we investigated the distance between zoomorphic objects and either animals or objects (Fig. 3c), as calculated in the previous results section. The ANOVA with Distances (Animal < Zoomorphic; Object < Zoomorphic) x Network revealed significant interaction terms for both alternative DNNs (FT Animacy DNN: *F*(2, 64) = 94.88, *p* < 0.001; FT Face-body-object DNN: *F*(2, 64) = 73.33, *p* < 0.001). Post-hoc analyses showed that relative to the pretrained ImageNet DNN, the distance between animals and zoomorphic objects (*M* = 1.18) even increased in both FT Animacy DNN (*M* = 1.62) and FT Face-body-object DNN (*M* = 1.52), although only in the former it reached statistical significance (*t*(32) = 3.01, *pcorr* = 0.006, *BF*_10_ = 30; *t*(32) = 0.71, *pcorr* = 0.56, *BF*_10_ = 2.5, respectively). At the same time, compared to PT ImageNet DNN, the distance between zoomorphic and regular objects (*M* = 0.77) significantly decreased (FT Animacy DNN: *M* = 0.38, *t*(32) = −6.52, *pcorr* < 0.001, *BF*_10_ = 21; FT Face-body-object DNN: *M* = 0.38, *t*(32) = −5.79, *pcorr* < 0.001, *BF*_10_ = 15). These findings were significantly opposed relative to representations observed in FT Animal bias DNN, such that in both DNNs (FT Animacy and FT Face-body-object) the distance between animals and zoomorphic objects was significantly larger (FT Animacy: *M* = 1.62, *t*(32) = 8.68, *pcorr* < 0.001, *BF*_10_ = 1.4 × 10^9^; FT Face-body-object: *M* = 1.52, *t*(32) = 6.01, *pcorr* < 0.001, *BF*_10_ = 2.8 × 10^6^), whereas zoomorphic images were represented significantly closer to real objects (FT Animacy: *M* = 0.38, *t*(32) = −7.17, *pcorr* < 0.001, *BF*_10_ = 4.1 × 10^5^; FT Face-body-object: *M* = 0.38, *t*(32) = −7.50, *pcorr* < 0.001, *BF*_10_ = 2.6 × 10^5^).

In terms of correlations with the neural representations (Fig. 3d), none of the alternative training regimes (Animacy and Face-Body) resulted in a representational structure that could reach performance obtained by the Animal bias DNN. Specifically, for both alternative DNNs, an ANOVA with factors ROI (EVC; posterior VTC; anterior VTC) and Network (FT Animal bias bias; PT ImageNet; FT Animacy or FT Face-body-object) on the correlations revealed a significant interaction between ROI and Network (FT Animacy: *F*(4, 60) = 8.05, *p* = 0.002; FT Face-body-object: *F*(4, 60) = 11.05, *p* < 0.001). Most importantly, in each case, the alternative DNN showed significantly lower correlations in ant VTC compared to FT Animal bias (FT animacy: *r*_*AnimacyDNN*_ = 0.09, *r*_*AnimalbiasDNN*_ = 0.18, *t*(15) = −4.83, *pcorr* < 0.001; FT Face-body-object: *r*_*Face*−*body*−*objectDNN*_ = 0.06, *r*_*AnimalbiasDNN*_ = 0.18, *t*(15) = −6.84, *pcorr* < 0.001), while correlations were similar to the ones observed for PT ImageNet (FT animacy: *r* = 0.08, *t*(15) = 1.43, *p* = 0.20; FT Face-body-object: *r* = 0.08, *t*(15) = −2.15, *pcorr* = 0.09). To conclude, fine-tuning the network to selectivity for animacy or for faces and bodies captured less of the anterior VTC representations than the DNN fine-tuned to Animal bias, and did not improve the fit relative to the standard pretrained DNN.

### Hypothesis two: Can the Animal bias emerge in DNNs trained to focus on shape over texture?

Geirhos and colleagues^51^ found that DNNs tend to rely more upon texture than shape when categorizing objects (texture bias), while human observers show the opposite bias (shape bias). Given that this finding is one of the best documented discrepancies between human and AI vision, we considered the possibility that the Object bias in DNNs might be related to their texture bias, reducing the two seemingly separate phenomena to one and the same underlying tendency. Geirhos and colleagues^51^ also showed that the texture bias in DNNs is converted into a shape bias when using a different training regime with a stimulus set in which images are more variable in texture and style, the so-called Stylized ImageNet. We tested to which extent a DNN pretrained on the Stylized ImageNet (PT Stylized ImageNet) would induce an Animal bias.

**Fig. 4:**
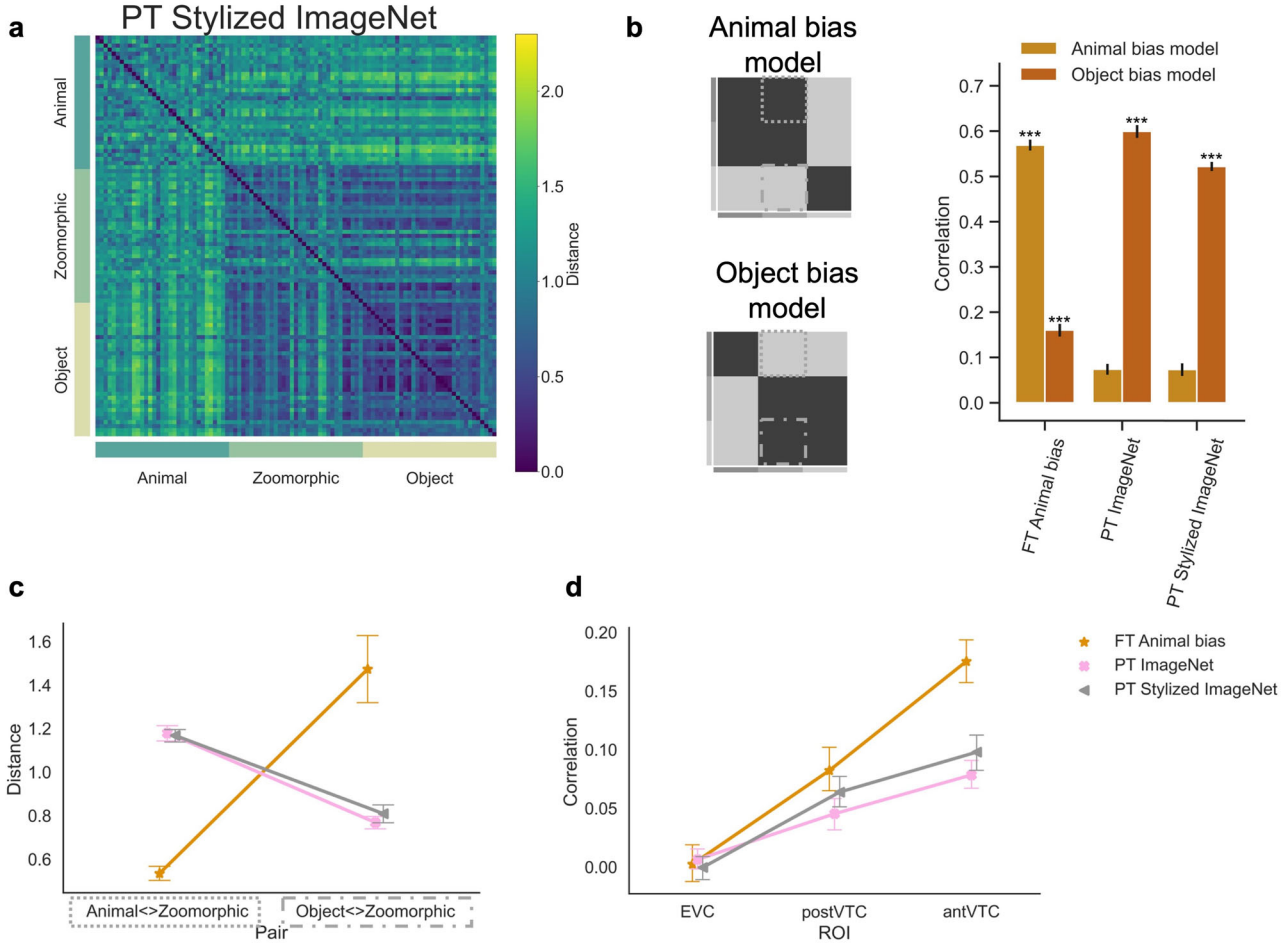
Alternative hypothesis two: tackling shape-texture bias. **a** The pretrained Stylized ImageNet Representational Similarity Matrix included in the analyses is displayed. PT = pretrained AlexNet, FT = fine-tuned AlexNet. **b** Graphical illustration of the independent Animal and Object bias model. The graph represents the correlation for each neural network with each bias model. Significant values (i.e., ***p < 0.0001, **p < 0.001, *p < 0.01), were computed with permutation tests (10,000 randomizations), and error bars indicate standard error computed via bootstrapping. **c** Mean distance score of each image in the contrast Animal < > Zoomorphic and Object < > Zoomorphic and this for each neural network. For violin plots showing the full distributions, please see Supplementary Fig. 4. **d** Graph with the individual correlations between the neural data in the three regions of interest (i.e., EVC, posterior- and anterior VTC) and the neural network data resulting from the different training regimes. All error bars represent the standard error.

Findings showed (Fig. 4b) a positive correlation between the PT Stylized ImageNet DNN and the independent Object bias model (*r* = 0.52) which was significantly higher relative to the correlation observed with the Animal bias model (*r* = 0.07, *p* < 0.001). Analyzing the representational distance (Fig. 4c) for zoomorphic objects relative to the animals and the objects revealed a representational space like the one observed in the baseline pretrained ImageNet DNN. That is, animals and zoomorphic objects in the PT Stylized DNN (*M* = 1.17, significant interaction effect *F*(2, 64) = 106.85, *p* < 0.001) showed a larger distance compared to FT Animal bias (*M* = 0.53, *t*(32) = 19.36, *pcorr* < 0.001, *BF*_10_ = 3.4 × 10^18^) but a similar distance relative to PT ImageNet (*M* = 1.18, *t*(32) = −0.04, *pcorr* = 0.97, *BF*_10_ = 0.26). Conversely, there was a smaller distance between zoomorphic and real objects in PT Stylized (*M* = 0.81) compared to FT Animal bias (*M* = 1.47, *t*(32) = −4.29, *pcorr* < 0.001, *BF*_10_ = 2.2 × 10^3^) and an equal distance relative to PT ImageNet (*M* = 0.77, *t*(32) = −0.52, *pcorr* = 0.65, *BF*_10_ = 0.32). These findings illustrated the absence of a strong Animal bias in PT Stylized ImageNet.

Next, we looked at the correlation with neural representations (Fig. 4d). A repeated measures ANOVA with factors ROI (EVC; post VTC; ant VTC) x Network (PT Stylized ImageNet; PT ImageNet; FT Animal bias) on the correlation with the neural data revealed significant interacting terms (*F*(4, 60) = 7.99, *p* = 0.003). Post-hoc analyses showed that whereas in EVC there was no difference between the three DNNs (all *pcorr* > 0.36), when moving downstream, this difference increased and reached significance in ant VTC where correlations for the FT Animal bias DNN were significantly higher relative to the PT Stylized DNN (ant VTC: *t*(15) = 4.54, *pcorr* = 0.002) and the baseline PT ImageNet DNN (ant VTC: *t*(15) = 5.34, *pcorr* < 0.001). In addition, relative to the baseline PT ImageNet DNN, the PT Stylized DNN was slightly better correlated to the neural data in VTC but these differences did not survive correction for multiple comparisons (post VTC; *t*(15) = 2.71, *pcorr* = 0.036). To conclude, even a network that is trained to shift to a human-like shape bias does not show a human-like Animal bias.

### Hypothesis three: Animal bias because of network trained with more naturalistic categories

**Fig. 5:**
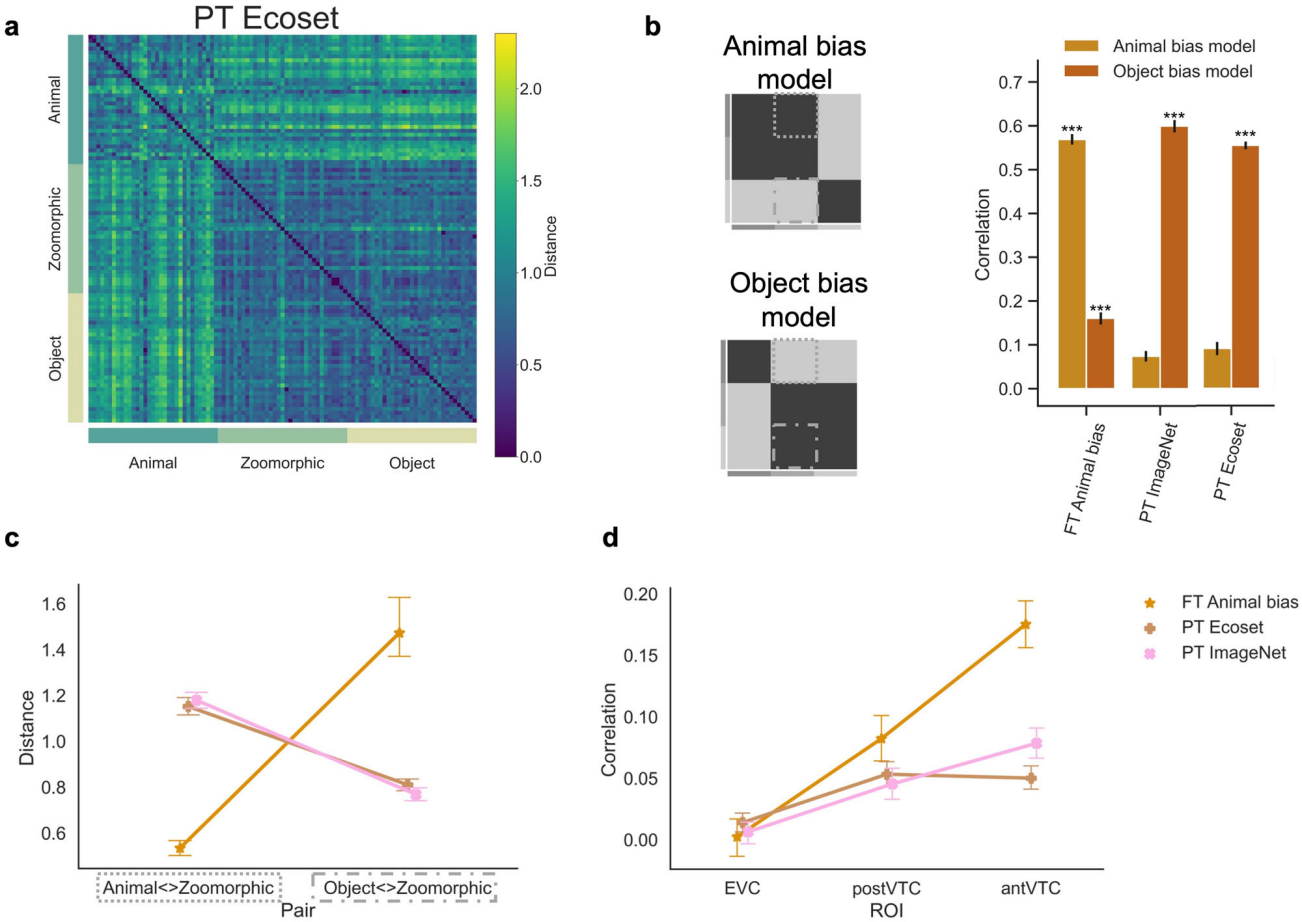
Alternative hypothesis three: training with ecological categories. **a** The Representational Similarity Matrix of the pretrained Ecoset network included in the analyses is displayed. PT = pretrained AlexNet, FT = fine-tuned AlexNet. **b** Graphical illustration of the independent Animal and Object bias model. The graph represents the correlation for each neural network with each bias model. Significant values (i.e., ***p < 0.0001, **p < 0.001, *p < 0.01), were computed with permutation tests (10,000 randomizations), and error bars indicate standard error computed via bootstrapping. **c** Mean distance score of each image in the contrast Animal < > Zoomorphic and Object < > Zoomorphic and this for each neural network. For violin plots showing the full distributions, please see Supplementary Fig. 4. **d** Graph with the individual correlations between the neural data in the three regions of interest (i.e., EVC, posterior- and anterior VTC) and the neural network data resulting from the different training regimes. All error bars represent the standard error.

Furthermore, we tested the hypothesis that training with more ecologically valid categories would result in a human-like Animal bias. A previous study introduced the ‘Ecoset’ stimulus set and showed that training with Ecoset results in a representational structure in later DNN layers that fits slightly better with brain representations compared to training with ImageNet^52^. We tested the effect of this Ecoset training on the representation of zoomorphic objects by testing AlexNet pretrained with Ecoset. Figure 5a displays the RDM obtained for this PT Ecoset DNN, alongside the previously reported RDMs for PT ImageNet and FT Animal bias. The visual similarity between PT Ecoset and PT ImageNet is striking.

In quantitative analyses, we computed the correlation with the conceptual models (Fig. 5b) and observed positive correlations with both models but much more convincingly with the Object bias model (*r* = 0.56) than with the Animal bias model (*r* = 0.09, *p*_*paired*_ < 0.001).

Second, the Animal < Zoomorphic and Object < Zoomorphic distances (Fig. 5c) of the stimuli were investigated in an ANOVA with interacting factors Distances (Animal < Zoomorphic; Object < Zoomorphic) and Network (PT Ecoset; PT ImageNet; FT Animal bias). As for the previous results sections, a significant interaction effect was found (*F*(2, 64) = 118.16, *p* < 0.001) and further analyzed in post-hoc analyses. Results showed that animals and zoomorphic representations were farther apart in PT Ecoset DNN (*M* = 1.15) relative to FT Animal bias DNN (*M* = 0.53, *t*(32) = −16.43, *pcorr* < 0.001, *BF*_10_ = 1.7 × 10^15^), but similar to PT ImageNet (*M* = 1.18, *t*(32) = −0.95, *pcorr* = 0.404, *BF*_10_ = 0.28). Further, the distance between zoomorphic and real objects was much smaller in PT Ecoset DNN (*M* = 0.81) compared to FT Animal bias DNN (*M* = 1.47, *t*(32) = −4.00, *pcorr* < 0.001, *BF*_10_ = 3.9 × 10^3^) but did not differ from PT ImageNet (*M* = 0.77, *t*(32) = 2.46, *pcorr* = 0.025, *BF*_10_ = 0.4). These results reveal that zoomorphic objects were represented more like real objects than like animals in the Ecoset pretrained DNN, in line with the Object bias observed in the standard pretrained ImageNet DNN.

Third, the correlation with the neural data (Fig. 5d) was investigated and similar results to PT ImageNet DNN were observed. A repeated measures ANOVA with factors ROI (EVC; post VTC; ant VTC) x Network (PT Ecoset; PT ImageNet; FT Animal bias) revealed a significant interaction effect (*F*(4, 60) = 15.00, *pcorr* < 0.001), which was further analyzed post-hoc. In anterior VTC, PT Ecoset DNN (*r* = 0.05) correlated significantly less compared to FT Animal bias (*r* = 0.18, *t*(15) = −7.41, *pcorr* < 0.001) and to PT ImageNet DNN (*r* = 0.08, *t*(15) = −4.03, *pcorr* = 0.003). In posterior VTC, no significant differences were found between PT Ecoset DNN (*r* = 0.05) and FT Animal bias DNN (*r* = 0.08, *t*(15) = −1.77, *pcorr* = 0.17) nor with PT ImageNet DNN (*r* = 0.05, *t*(15) = 1.47, *pcorr* = 0.24). To conclude, pretrained Ecoset DNN was not able to capture more of the representations of zoomorphic objects in ant VTC than standard pretrained DNNs and performed even worse.

### Hypothesis four: Animal bias due to recurrent processing

The architecture of the human brain is characterized by an abundance of recurrent connections. These connections might be fundamental for understanding neural processing, for example as the primary substrate for predictive coding^53–55^ or for perceiving illusory contours^56^. We hypothesized that the way objects are represented, and in the current context more specifically how zoomorphic objects, might be influenced by the presence of recurrent processing. We trained and tested a recurrent version of AlexNet based upon the principles of predictive coding, recurrent PT ImageNet^57^.

**Fig. 6:**
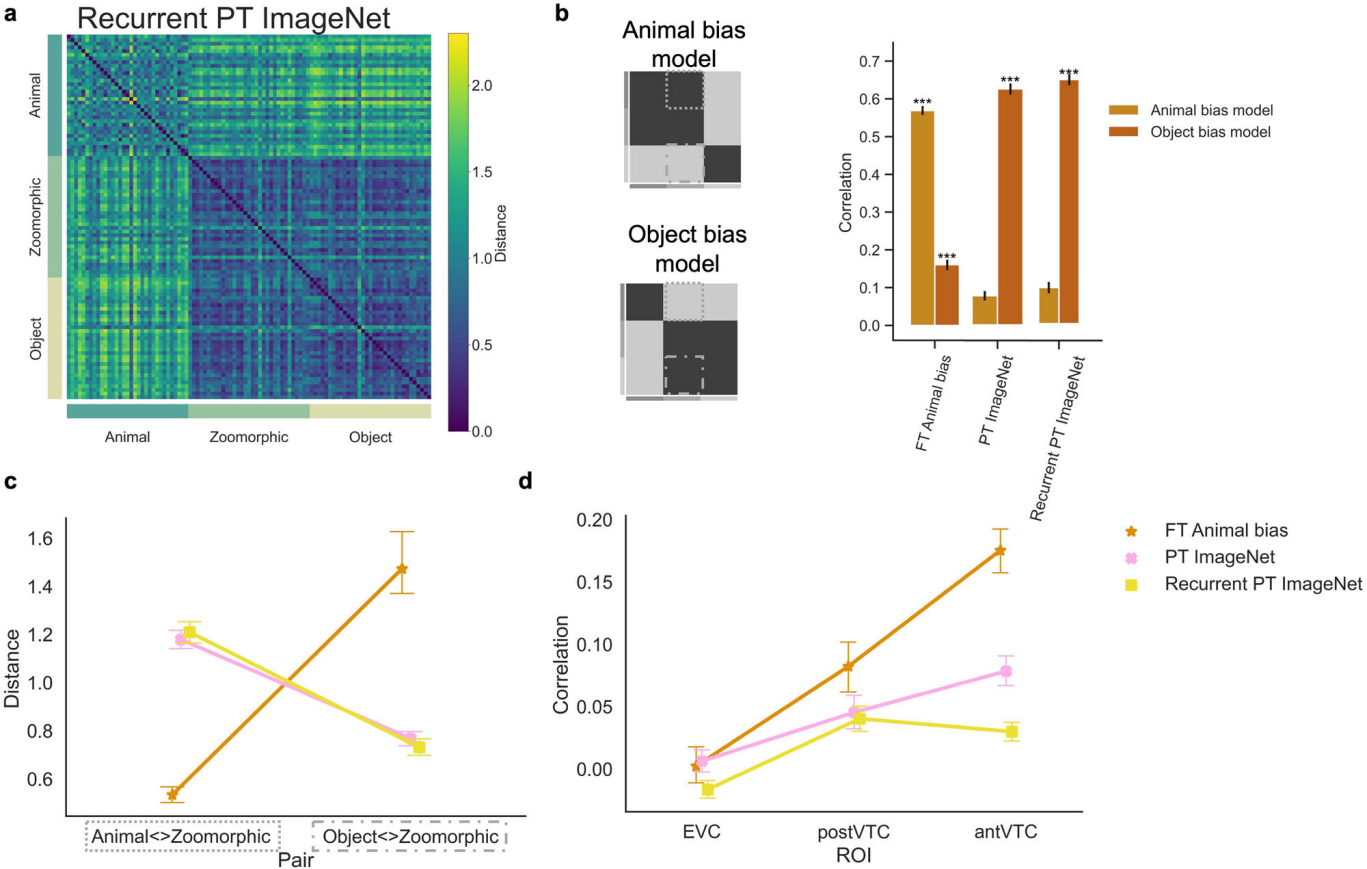
Alternative hypothesis four: recurrence. **a** The Representational Similarity Matrix of the recurrent PT ImageNet included in the analyses is displayed. PT = pretrained AlexNet, FT = fine-tuned AlexNet. **b** Graphical illustration of the independent Animal and Object bias model. The graph represents the correlation for each neural network with each bias model. Significant values (i.e., ***p < 0.0001, **p < 0.001, *p < 0.01), were computed with permutation tests (10,000 randomizations), and error bars indicate standard error computed via bootstrapping. **c** Mean distance score of each image in the contrast Animal < > Zoomorphic and Object < > Zoomorphic and this for each neural network. For violin plots showing the full distributions, please see Supplementary Fig. 4. **d** Graph with the individual correlations between the neural data in the three regions of interest (i.e., EVC, posterior- and anterior VTC) and the neural network data resulting from the different training regimes. All error bars represent the standard error.

The RDM in Fig. 6a displays a very similar similarity structure as found in the feedforward AlexNet. Statistical analyses revealed that the object bias model correlated significantly with the recurrent PT ImageNet RDM (*r* = 0.60, *p* < 0.001), while the animal bias model mostly failed (*r* = 0.09, *p* = 0.012; see Fig. 6b). In addition, an ANOVA with factors Distances and Network (recurrent PT ImageNet; PT ImageNet; FT Animal bias) on the stimulus distances Animal < Zoomorphic and Object < Zoomorphic (Fig. 6c) demonstrated a significant interaction effect (*F*(2, 64) = 115.54, *p* < 0.001). Post-hoc analyses showed that the distance between animal and zoomorphic objects was similar between recurrent PT ImageNet (*M* = 1.21) and its feedforward counterpart PT ImageNet (*M* = 1.18, *t*(32) = 0.37, *p* = 0.71, *BF*_10_ = 0.28), and much larger than in FT Animal bias (*M* = 0.53, *t*(32) = 17.46, *p* < 0.001, *BF*_10_ = 1.1 × 10^15^). The same clustering of networks was found for the distance between zoomorphic and regular objects, where the distance was again similar for recurrent PT ImageNet (0.73) and PT ImageNet (0.77; *t*(32) = 1.07, *p* = 0.36, *BF*_10_ = 0.33), and now much smaller in recurrent PT ImageNet than in FT Animal bias (1.47; *t*(32) = −4.88, *p* < 0.001, *BF*_10_ = 2.4 × 10^4^). These tests confirm that recurrent PT ImageNet is characterized by an Object bias that is very similar to the feedforward AlexNet model (PT ImageNet).

Last, recurrent PT ImageNet did not show a better correlation with the neural data (Fig. 6d) than (feedforward) PT ImageNet. A repeated measures ANOVA with factors ROI (EVC; post VTC; ant VTC) x Network (recurrent PT ImageNet; PT ImageNet; FT Animal bias) demonstrated a significant interaction effect (*F*(4, 60) = 13.31, *pcorr* < 0.001), which was further analyzed post-hoc. In anterior VTC, recurrent PT ImageNet (*r* = 0.03) correlated significantly less than FT Animal bias (*r* = 0.18, *t*(15) = 8.11, *pcorr* < 0.001) and even less than PT ImageNet DNN (*r* = 0.05, *t*(15) = 5.41, *pcorr* < 0.001). In posterior VTC, there were no significant differences observed between recurrent PT ImageNet (*r* = 0.04) and FT Animal bias (*r* = 0.08, *t*(15) = 2.22, *pcorr* = 0.07) nor with PT ImageNet (*r* = 0.05, *t*(15) = 0.45, *pcorr* = 0.74).

### Hypothesis five: Animal bias in a language-informed network

Finally, this study investigated whether the Animal bias could be reproduced by an alternative approach that involves semantics or language^58,59^. Language-informed visual encoders and in particular the CLIP model of Open.AI have been shown recently to provide a better fit to tuning in visual cortex compared to vanilla AlexNet^60^. The effect of such a model on the representation of zoomorphic objects was evaluated through the encoding layer of the CLIP model, which was identical to Wang and colleagues^60^.

**Fig. 7:**
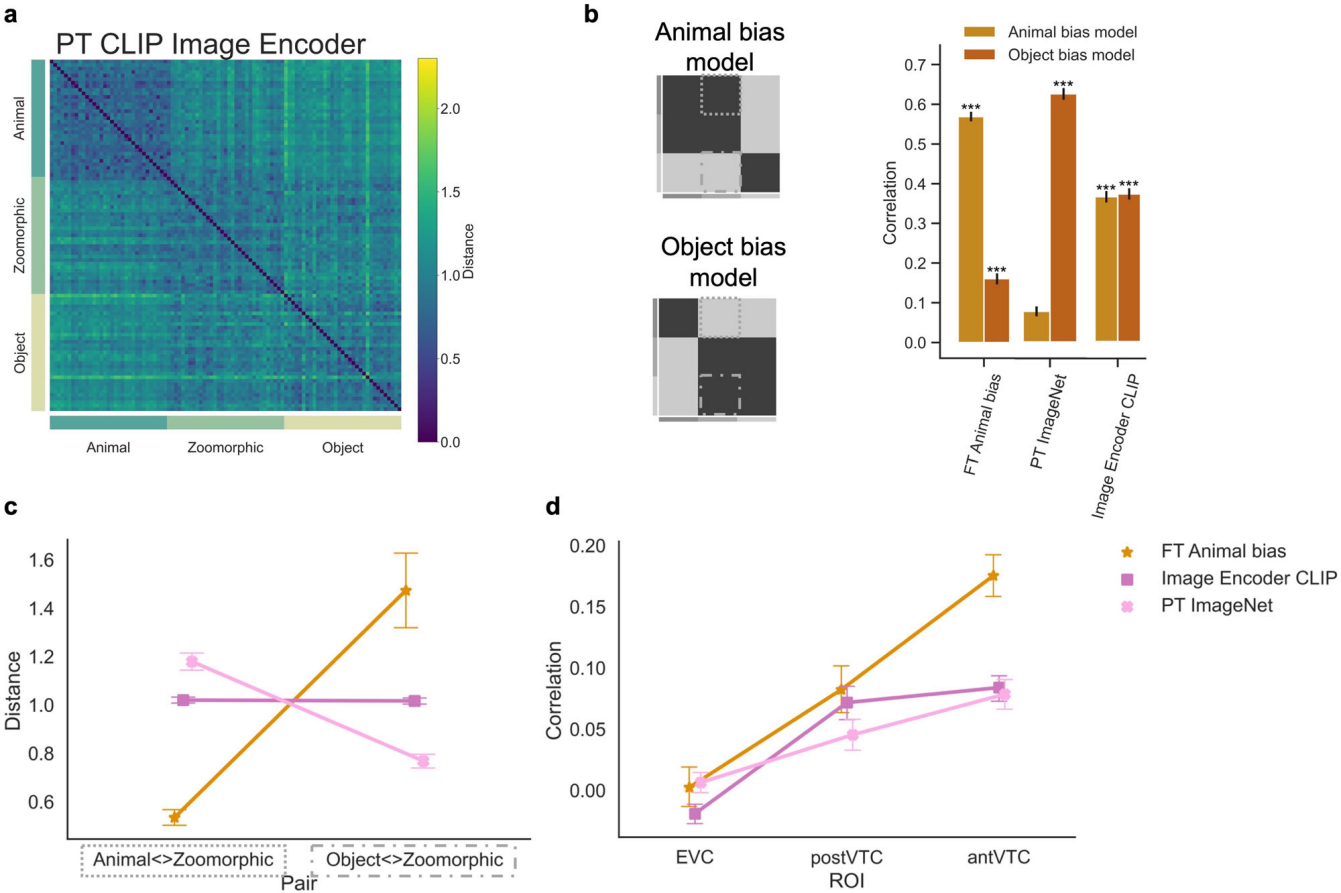
Alternative hypothesis five: language-informed model. **a** The Representational Similarity Matrix of the CLIP network included in the analyses is displayed. PT = pretrained AlexNet, FT = fine-tuned AlexNet. **b** Graphical illustration of the independent Animal and Object bias model. The graph represents the correlation for each neural network with each bias model. Significant values (i.e., ***p < 0.0001, **p < 0.001, *p < 0.01), were computed with permutation tests (10,000 randomizations), and error bars indicate standard error computed via bootstrapping. **c** Mean distance score of each image in the contrast Animal < > Zoomorphic and Object < > Zoomorphic and this for each neural network. For violin plots showing the full distributions, please see Supplementary Fig. 4. **d** Graph with the individual correlations between the neural data in the three regions of interest (i.e., EVC, posterior- and anterior VTC) and the e=neural network data resulting from the different training regimes. All error bars represent the standard error.

The RDM in Fig. 7a suggests that there is more confusion compared to standard pretrained ImageNet on the representation of zoomorphic objects as either being closer to real animals or to objects. Statistical analyses confirmed that both the independent animal and object bias model significantly (*p* < 0.001) correlated (animal bias: *r* = 0.36; object bias: *r* = 0.37) with the CLIP RDM results (Fig. 7b). Furthermore, the stimulus distances Animal < Zoomorphic and Object < Zoomorphic (Fig. 7c) were analyzed in an ANOVA with factors Distances and Network (Image Encode CLIP; PT ImageNet; FT Animal bias). Findings revealed a significant interaction effect (*F*(2, 64) = 116.23, *p* < 0.001), which was further investigated by means of post-hoc analyses. The distance between animal and zoomorphic conditions was greater in Image Encoder CLIP DNN (*M* = 1.02) compared to FT Animal bias DNN (*M* = 0.53, *t*(32) = 13.32, *pcorr* < 0.001, *BF*_10_ = 9.6 × 10^17^), but closer together in CLIP DNN relative to PT ImageNet DNN (*M* = 1.18, *t*(32) = −3.41, *pcorr* = 0.002, *BF*_10_ = 2.1 × 10^2^). The reverse was true for the distance between zoomorphic and object conditions, such that it was smaller in CLIP DNN (*M* = 1.02) compared to FT Animal bias DNN (*M* = 1.47, *t*(32) = −2.06, *pcorr* = 0.048, *BF*_10_ = 38) and larger relative to PT ImageNet DNN (*M* = 0.77, *t*(32) = 7.04, *pcorr* < 0.001, *BF*_10_ = 4.1 × 10^7^). These results were somehow different from previous alternative approaches, such that the CLIP DNN did not have an Object bias, nor an Animal bias for zoomorphic objects. More specifically, these objects were represented as much as real objects as like animals.

Last, the CLIP DNN correlated in a similar fashion with the neural data (Fig. 7d) as was the case for PT ImageNet DNN. A repeated measures ANOVA with factors ROI (EVC; post VTC; ant VTC) x Network (Image Encoder CLIP; PT ImageNet; FT Animal bias) revealed a significant interaction effect (*F*(4, 60) = 8.46, *pcorr* = 0.001), which was further analyzed post-hoc. In anterior VTC, CLIP DNN (*r* = 0.08) correlated significantly less than FT Animal bias (*r* = 0.18, *t*(15) = 5.07, *pcorr* = 0.001) and to the same degree as PT ImageNet DNN (*r* = 0.05, *t*(15) = 0.41, *pcorr* = 0.773). In posterior VTC, there were no significant differences observed between CLIP DNN (*r* = 0.07) and FT Animal bias DNN (*r* = 0.08, *t*(15) = 0.69, *pcorr* = 0.648) nor with PT ImageNet DNN (*r* = 0.05, *t*(15) = 2.03, *pcorr* = 0.136). As for the other alternative models, Image Encode CLIP could not grasp the zoomorphic object representations in anterior VTC like acquired by FT Animal bias DNN, and resembled the standard pretrained DNN in this matter. These results confirm the superiority of the Animal bias model in capturing high-level representations in visual cortex.

Overall, these findings with the CLIP model illustrate that even the constraint to merge vision with language does not result in an Animal bias as it is found in human visual cortex. Nevertheless, the representations of zoomorphic objects in CLIP, with an absence of an Object bias as in many other DNN instantiations, illustrate that CLIP captures the ambiguous nature of these animal-like objects to some degree. The integration with language allows us to relate these representations in linguistic terms. CLIP was used as a basis for a model to generate image captions of images, CLIPCAP^61^. We anecdotally tested CLIPCAP by presenting it with the picture of the cow-shaped cup. The resulting caption provided by the model was “A black and white photo of a coffee cup with a cow on it”. This is a good description of the zoomorphic nature of the depicted objects. Of the nine zoomorphic objects in the set of Bracci et al.^13^, four were recognized as zoomorphic. For seven, the description referred to an animal as well as an object item, but sometimes without explicitly recognizing the zoomorphic meaning (e.g., “A large owl is sitting on a table”).

## Discussion

In this work, we followed a computational approach to understand the tendency of the human visual system to represent zoomorphic objects as real animals. We investigated whether and under which conditions a training protocol in DNNs can shift the representation of zoomorphic objects towards animals rather than objects. Training a DNN to explicitly categorize zoomorphic objects as animals increased the similarity to the Animal bias found in the brain. Obtaining such a computational Animal bias model allowed us to test a variety of other training protocols motivated by properties of human object perception. Only the explicit Animal bias training showed a shift towards an Animal bias model and by far the most convergence with anterior VTC representations. These are not just small quantitative differences, but findings that point to a representation of a different nature.

There are many reasons to be very optimistic about the abilities of ImageNet-trained networks to perform object classification tasks and the convergence of these networks with perceptual and neural object representations^62,63^. In addition, researchers were rightfully impressed by further improvements due to adjusted training protocols, e.g., by addressing texture bias^51^, including more ecologically valid stimulus sets^52^ and recurrent processing^37,57^. Yet all these previous developments resulted in networks with tendencies opposite to human representations when it comes to the representation of zoomorphic objects. First, an Animal bias does not seem to be related to the strong selectivity for animacy and for faces and bodies in the human brain^10^. These distinctions are not included in the standard ImageNet training protocol, but we found that fine-tuning pretrained networks to these distinctions did not lead to a human-like Animal bias. Second, also a model that has a shape bias instead of texture bias^51^ still has an object bias instead of an Animal bias. Same goes for, as a third tested hypothesis, the use of a more ecologically valid stimulus set, Ecoset^52^, which did not result in an Animal bias either. Fourth, recurrent networks, who can capture interesting aspects of human information processing^44,46,56,57^, also do not seem to show a shift towards an Animal bias. This finding is consistent with recent findings^64^ with multivariate EEG that show that the Animal bias in the human ventral visual system is already observed in the early parts of the EEG response. Fifth and finally, even a model trained to connect images with text^60^, which might allow to capture potential effects of semantic image understanding on visual processing, did not show a human-like Animal bias. Interestingly, the CLIP model represented zoomorphic objects as being “in the middle” between animals and objects. This was further illustrated by the captions generated for images of zoomorphic objects by the CLIPCAP model. CLIP representations did no longer show the Object bias found in many other DNN instantiations. At the same time, CLIP still did not display an Animal bias as observed in human visual cortex.

These five hypotheses were reported thoroughly in this manuscript. In addition, we explored other possible alternatives. In particular, RDMs and correlations for some other alternative DNN training regimes, such as a face-trained AlexNet^65^, a video action recognition trained ResNet^66^, and an AlexNet trained with a contrastive learning algorithm^67^ are reported in the Supplementary material. None of these tests revealed an Animal bias.

Importantly, these findings extend beyond the specific models tested in our study. The Brain Score team independently evaluated a diverse collection of models from the Brain Score platform as part of the 2024 Brain Score competition (Neural Benchmark track). Their results corroborated our observations, revealing a consistent Object bias in the tested DNNs that contrasts sharply with the Animal bias observed in both our neural data and the FT Animal bias DNN. The stark difference between the model RDMs (exhibiting an Object bias) and the neural data (demonstrating a clear Animal bias) secured a second place in the competition, underscoring two critical points: (1) the effect is remarkably consistent across a wide range of tested networks, and (2) the discrepancy we observe between models’ representational geometry and brain data is substantial enough to be highly competitive in a benchmark designed to assess (the failure of) neural prediction accuracy. These results further reinforce the significance of our findings and highlight the unique nature of the Animal bias in human visual processing, which appears to be challenging for current DNN architectures and training paradigms to capture without explicit training.

Against all these models that fail to show an Animal bias, one neural network stands out by its ability to show an Animal bias and capture this aspect in the neural data: The FT Animal Bias model that was explicitly fine-tuned to cluster zoomorphic objects with animals. While the emergence of an Animal bias in the condition where zoomorphic objects and animals were treated as equivalent during training might seem intuitive, we argue that the success of this FT Animal Bias model is both significant and non-trivial. Firstly, it serves as a crucial positive control, demonstrating the feasibility of inducing an animal bias in DNNs and providing a benchmark for our other experimental conditions. Secondly, the success of this condition relies on the network’s ability to generalize to novel exemplars, a key aspect of human-like categorization that is not guaranteed in DNNs. Moreover, the degree of alignment with human neural representations was not predetermined, offering quantitative insights into the potential mechanisms underlying perceptual biases. Lastly, this condition provides an important proof of principle, showing that DNNs *can* develop representational structures mirroring human perceptual biases under certain learning conditions. Thus, while this result may appear less surprising, it plays a central role in our investigation of the origins of the Animal bias.

Together, these findings suggest that explicit supervised training to classify zoomorphic objects as animals is a crucial condition to develop an Animal bias, as such providing an unprecedented understanding of the human perception of zoomorphic objects.

Even if direct exposure to zoomorphic objects is relevant, this finding leaves open several possibilities in terms of the exact learning mechanisms that might be involved. Effects found through supervised learning can also result from unsupervised protocols. For example, Konkle and Alvarez^67^ showed that in fully self-supervised models trained on individual images, categories, and hierarchical features alike those in the ventral visual stream emerged as observed in category-supervised models. The same might be true for human development, or, even more likely, a mixture of supervised and unsupervised learning might occur. The high frequency with which human infants encounter zoomorphic objects, in many cases even before they have seen the real animals, gives plenty opportunity for supervised as well as unsupervised learning mechanisms to mold the development of an Animal bias in the human visual system. Furthermore, what we model as a developmental training effect might for the real human visual system have emerged across an evolutionary time scale. A bias to, when in doubt, interpret an object as animate, might have proven important for survival to not miss potential predators or prey. Our computational findings are equally consistent with this evolutionary hypothesis. Further behavioral and neurophysiological work in infants and other primate species could shed light on this issue. Selectivity for animals versus non-animals has been found in infants^68^ as well as monkeys^69^, yet it is unknown how they represent zoomorphic objects and how this would relate to experience (e.g., age of the infants). For now, we tend to consider the developmental hypothesis as the most likely option given the massive effects of learning on category selectivity for faces^70^. However, future research could directly test the evolutionary versus developmental hypotheses about the emergence of an Animal bias by using alternative modeling approaches that can capture long-term evolutionary dynamics or simulate differential costs associated with animal recognition.

Our investigations illustrate the value of trained deep convolutional networks to investigate which properties of complex object representations go hand in hand and which do not. Neuroscientists and psychologists interested in vision have welcomed these computational models enthusiastically. These models can predict many properties of human visual performance and representations that remained elusive with earlier modeling approaches^25,27,28,71–73^. However, as emphasized recently by Bowers and colleagues^39^, a general correspondence can hide many fundamental differences if studies do not (i) include experiments that focus upon specific psychological or neural phenomena that target important properties of information processing systems, and (ii) investigate hypotheses about how these psychological phenomena come about. We implemented this approach. First, the Animal bias in the human visual system is a prime candidate of an important phenomenon, given the emphasis upon the animacy dimension in human vision^74^ and the marked discrepancy in how humans and vanilla DNNs represent ambiguous zoomorphic objects^13^. Second, we investigated a wide range of hypotheses about which mechanisms and learning experience might underlie the human Animal bias.

In sum, the tendency to represent zoomorphic objects as animals is a particularly prominent feature of human object processing that is only observed in neural networks when the similarity between zoomorphic objects and animals is emphasized explicitly by the training regime. Our computational analyses show that this human animal bias stands apart as a separate phenomenon that does not piggyback on other known causes of human-unique aspects of visual object cognition.

## Methods

### Stimuli

This study comprised different stimulus sets, collected from different sources (Fig. 1a). Here we only describe the stimulus sets compiled specifically for this study (Fig. 1b), in the section on DNN training regimes we will rely upon additional stimulus sets available in the literature. There were five main stimulus categories: animals, objects, zoomorphic objects, faces, and bodies. For the categories of animals, objects, and human faces, the Caltech-256 stimulus set^75^ was used, which has 257 categories, with on average 119 images per category. For each category (i.e., animal, object, human face), we looked for the Caltech categories that fell under this category label. In a next step, all the different images from these Caltech categories were stored as one category (e.g., animals). As a result, our main categories did not contain subcategories (e.g., different animals), but one big set of all images (e.g., animals) together. The animal faces subcategory were a random sample of the Animal Faces-HQ stimulus set^76^. The remaining categories (zoomorphic objects and bodies) were the result of a google search with the use of the Image Downloader Chrome Extension to download the images in bulk, which were later manually evaluated whether they matched our search criteria.

For training and fine-tuning (FT) the neural networks, we created different stimulus sets that involve a subset of categories or combination of categories (Fig. 6a). First, the FT Animal bias set consisted of two categories, namely objects and a combination of fifty-fifty animal and zoomorphic images put together as one category. Second, the FT Object bias contained the same images as FT Animal bias but organized here as a pure animal category and a mixed category with objects and zoomorphic objects. Third, FT Animacy had two categories, namely animals and objects, which did not contain any zoomorphic images. Last, FT Face-body-object consisted of three categories: faces (animal + human), bodies (animal + human), and objects. To train and validate the networks of interest, all stimulus sets created here, were further split into a training (‘train’) and validation (‘val’) folder (Fig. 1c).

Another and last collection of stimuli was created to test our DNNs (Fig. 1c). Importantly, none of these images were part of training or validating the networks and were only used to evaluate our hypotheses (see Results). This collection consisted of three categories: animals, zoomorphic objects, and objects. Each category contained 33 images, of which nine were identical to the stimuli used in the study by Bracci and colleagues^13^. This subset with nine items was used to correlate the DNN data with the neural data from Bracci and colleagues^13^.

### DNN data

Convolutional deep neural networks have been trained in object classification tasks, in which they reach very high levels of proficiency even with only a ‘small’ number of layers^62^. AlexNet, which won the ILSVCR2012 with the complexity of ‘only’ eight layers, is the first exemplar of this generation of networks and since its conception also the one that has been used most in comparisons with human behavior and neuroscience^62^. AlexNet is due to its limited complexity and good object recognition performane is an ideal candidate for this study. Of particular interest for our purposes are the later/deeper layers, also called fully connected layers, as these act as a decoder after the encoding non-linear layers, being therefore well suited for object classification^77–80^, and have been shown to correspond best to representations found in human ventral visual cortex^28^. In Supplementary Fig. 1, we show that our findings do not critically depend on this architecture or layer, given that we replicated the main findings with another architecture (VGG) and for layer FC7 (Supplementary Fig. 2).

To investigate the role of recurrent processing, we employed the Predify framework^57^ to incorporate predictive coding dynamics into our pretrained AlexNet architecture. Predictive coding^53,55^ is a computational framework that posits that the brain actively generates predictions about the sensory input and uses these predictions to guide perception and inference via an iterative process. The Predify framework enables the integration of predictive coding dynamics into a feedforward network by representing the activity of each layer at a given time as a linear combination of four terms: feedforward drive, feedback, error correction, and memory. The feedforward drive represents the activations for the incoming sensory input, while the feedback acts a correction signal to minimize the prediction (i.e., reconstruction) error over timesteps. Error correction is the mechanism that adjusts the predictions made by each layer based on the feedback it receives from the layer above, and memory is responsible for maintaining a temporal consistency of the input. Specifically, the recurrent PT ImageNet started from the baseline (feedforward) AlexNet trained on ImageNet (PT ImageNet). The recurrent PT ImageNet network was constructed by incorporating recurrent connections following equations previously described in Alamia and colleagues^81^ and in Choksi and colleagues^57^. In broad terms, each feedforward layer (encoder) of the PT_ImageNet model was complemented with a feedback layer (decoder) dedicated to performing reconstruction tasks. The recurrent network is therefore composed of three encoding layers and three corresponding decoding layers, each with a distinct set of parameters. An encoding layer refers to a group of PyTorch layers defined as a sequential combination of convolutional (Conv2d) and activation (ReLU) layers, and it is followed by a pooling (MaxPool2d) layer.

To test the effect of learning to integrate visual representations with language, we also adopted the CLIP model as introduced by Open.AI^82^. We specifically focused upon the last visual encoding layer in this model, following Wang and colleagues^60^ who demonstrated that representations in this layer provide a better fit to tuning in visual cortex compared to AlexNet and other available models. The CLIP model utilizes a combination of image and text data during training, which enables it to learn a more comprehensive and contextually rich representation of the visual world. By leveraging the CLIP model, we were able to investigate whether and how the integration of visual and linguistic information might contribute to the observed effects in object recognition tasks and provide a deeper understanding of the relationship between language and visual perception in human cognition.

### DNN training regimes

We included multiple training regimes, resulting in multiple DNNs that share the same architecture but have a different training history. We must make a distinction between pretrained networks and fine-tuned networks. Pretrained networks were trained from scratch on a very large stimulus set, in this case ImageNet, Stylized ImageNet, or Ecoset. ImageNet consists of 1.2 million images, divided over 1000 image categories, of which 40% animals and 60% objects. Stylized ImageNet (SIN) was created by Geirhos and colleagues^51^ to limited to texture bias present in DNNs. All images in this dataset were transformed for the local texture cues to be useless in object categorization. The results reported by Geirhos et al. for AlexNet demonstrate a significant shift in bias after SIN training, where AlexNet trained on ImageNet showed 57.1% texture decisions vs. 42.9% shape decisions, while AlexNet trained on SIN exhibited approximately 23% texture decisions vs. 73% shape decisions. With Ecoset, Mehrer and colleagues^52^ constructed a stimulus set (i.e., 565 categories) in which the distribution of concepts is more ecologically valid (i.e., frequent in language and concrete) for human daily life, which in turn yielded slightly better models of human vision. The pretrained DNNs were not trained inhouse, but rather collected from the internet: pretrained AlexNet on ImageNet via PyTorch, Stylized ImageNet through the Geirhos GitHub page (https://github.com/rgeirhos/texture-vs-shape), and PT Ecoset through the Kietzmann lab website (https://www.kietzmannlab.org/Ecoset). The baseline pretrained network is trained on ImageNet (PT ImageNet), whereas Stylized ImageNet (PT Stylized ImageNet) was included to tackle the texture bias common in DNNs, and Ecoset (PT Ecoset) was the ecologically valid alternative that better matched models of human vision.

The Recurrent PT ImageNet was also trained on ImageNet. The feedforward weights were initially pretrained for image classification in a supervised manner (i.e., PT_ImageNet), while the feedback layers were subsequently added to the feedforward backbone network and optimized for a prediction (i.e., reconstruction) objective in an unsupervised fashion to minimize the reconstruction errors in each decoding layer. The primary goal of this dual-objective paradigm was to assess the potential benefits of integrating predictive coding dynamics as a feedback pathway into an existing state-of-the-art feedforward network. This method allowed us to concentrate on the added value provided by the feedback pathway when introduced to a pre-existing state-of-the-art feedforward network, and to make the feedforward and recurrent networks’ results more comparable. To train the feedback layers, we first froze all the pretrained feedforward layers, and then proceeded to train only the feedback layers on the ImageNet dataset. The training involved reconstructing all images in the dataset over 10 additional epochs, using a batch size of 64, a weight decay of 5e−4, Adam optimizer, and Mean Squared Error Loss function. We utilized a differential learning rate for each decoding layer, specifically, 1e−4, 1e−3, and 1e−3. Furthermore, we set the layer-dependent balancing coefficients (*β*_*n*_, *λ*_*n*_, and *α*_*n*_) for feedforward, feedback, and error-correction terms to 0.3, 0.3, and 0.01 during training, and to 0.3, 0.1, and 0.1 during testing. The total number of internal recurrent iterations (also referred to as timesteps) of the network was fixed at 5, which allowed for one feedforward pass of the input image and four subsequent recurrent iterations of the network. Image vectors were extracted during inference at the 5th timestep.

The baseline PT ImageNet served as the starting point for our fine-tuned networks, in which we further trained the PT ImageNet with our different training stimulus sets (Fig. 1b): animals + zoomorphic  < > objects (FT Animal bias), animals  < > zoomorphic + objects (FT Object bias), animals  < > objects (FT Animacy), and faces  < > bodies  < > objects (FT Face-body-object). This fine tuning is also known in the literature as transfer learning, and it makes use of a well-established trained network to further train the network on a similar, but smaller stimulus set. In this approach, the weights of most of the layers are not changed. This approach is beneficial when using a small but similar stimulus set as the early layers are well trained for basic image features and do not need to change because of the new training. The current study is interested in ‘deeper’ brain regions along the visual processing pathway, such as VTC. Given the small size of the stimulus set, all layers are set frozen except the penultimate fully connected layer (= FC7) and the last fully connected output layer (= FC8). FC8 is now characterized by the number of categories in the stimulus set (i.e., 2 or 3) rather than 1000 in pretrained (Stylized) ImageNet networks, and 565 in pretrained Ecoset. Fine-tuning was initialized with a learning rate of 1e−2, which was gradually multiplied by a factor of 0.1 in case the validation loss did not significantly improve over five consecutive epochs with a minimum learning rate of 1e−5. The training applied a batch size of 20 over 500 epochs, with a Stochastic Gradient Descent optimization algorithm, Cross Entropy Loss function, momentum of 0.9, and data is reshuffled at every epoch, resulting in a top-1 validation accuracy ranging between 90.9% and 99.5% (Table 1).

**Table 1:** Overview of the top-1 validation accuracies (%) for AlexNet for the different fine-tuned networks

Network	Validation accuracy (%)
FT Object bias	90.9
FT Animal bias	93.2
FT Animacy	93.1
FT Face-body-object	99.5

FT = fine-tuned

### DNN representational dissimilarity matrices

The test set with 99 images (i.e., 33 untrained images in each category: animal, zoomorphic, object) was loaded into the differently trained DNNs (i.e., PT ImageNet, PT Stylized ImageNet, PT Ecoset, FT Animal bias, FT Object bias, FT Animacy, and FT Face-body-object) to obtain the image representations from the output layer. These representations include the responses of all nodes in a layer and thus varied in size depending on the number of nodes/categories in the network output layer (i.e., respectively 1000, 1000, 565, 2, 2, 2, and 3 nodes). For each network, a representational dissimilarity matrix was constructed by calculating the distance score for each image pair (1−*R*_Pearson_). In the next step, these RDMs were normalized by dividing each score by the mean of the entire RDM.

A subset of the RDM, only containing the stimuli from Bracci and colleagues^13^, was used when correlating with the neural data available for that subset^13^. Additionally, during these analyses, these subset RDMs were limited to the upper triangle with exclusion of the diagonal.

The data obtained through DNNs are different from human data, as there is only one RDM per network (i.e., no individual data available like in human research). We applied non-parametric statistics for analyses that only entail DNN data. Permutation statistics across stimuli are used for correlation analyses, whereas more advanced non-parametric statistics for ANOVA were included to test multi-factorial models that consider variability across stimuli. More specifically, through the ARTool software^83^, the dependent variable (i.e., distance score) was transformed (i.e., align and rank) to perform a non-parametric factorial ANOVA and later post-hoc analyses, which are corrected for multiple comparisons (i.e., BH FDR). Bayes factors were calculated for each post-hoc comparison on the original (i.e., non transformed) data.

### Description of the data from Bracci and colleagues (2019)

Bracci and colleagues^13^ investigated the perceptual phenomenon ‘perceiving animacy’ in the human behavior and brain, and in DNNs. For this, they made use of independent models of animacy (hereinafter referred to as “Object bias model”) and appearance (hereinafter referred to as “Animal bias model”), which are in a sense RDMs containing zeros and ones for ideal cases where zero stands for 100% like each other and one for 0% similar. The Object bias model (Fig. 2b) expects great similarity along the category diagonal and between objects and zoomorphic objects, whereas the Animal bias model (Fig. 2b) expects great similarity between animals and zoomorphic objects next to the diagonal similarity. The RDMs coming from the behavioral, neural and DNN experiment were correlated with these independent model RDMs.

At the DNN level, two pretrained DNNs (i.e., VGG-19 and GoogLeNet) were evaluated with the Bracci test subset. Here a strong correlation was found with the Object bias model, whereas there was no sign of any correlation with the Animal bias model. In the neuroimaging part, subjects performed two different tasks in the scanner: an animacy task (“does this image depict a living animal?”; inducing an Object bias) and an appearance task (“does this image look like an animal?”; inducing an Animal bias). Bracci and colleagues et al.^13^ obtained RDM data from three regions of interest: early visual cortex (EVC), posterior (post-) and anterior (ant-) VTC. While EVC served as a control region of low-level features, VTC has shown its importance in object representation and categorization^84^. Results showed correlations in VTC with both models, but unexpectedly significantly stronger for the Animal bias model. Furthermore, when looking at post and ant VTC separately, results are even more pronounced in ant VTC where there is no longer a correlation with the Object bias model.

In our study, the new DNN data was correlated with the existing neural data. The neural RDMs consisted of an average across both tasks given that there was no effect of task in any of the ROIs.

## Supplementary information

SUPPLEMENTAL MATERIAL

reporting-summary

## Data Availability

The data used for final statistics are made available through the Open Science Framework at https://osf.io/7xcg9.

## Code availability

All DNN work, except for Ecoset, and analyses were done in PyTorch (v1.9.0) and Torchvision (v0.10.0) through Spyder (Python 3.8). For Ecoset, we used Code Ocean (https://codeocean.com/capsule/9570390/tree/v1) to extract the image vectors from the pretrained AlexNet on Ecoset. For conducting non-parametric factorial ANOVA and corresponding post-hoc analyses we used ARTool^83^. For generating the predictive coding recurrent DNN architecture we used the *predify* framework^57^. The code used for final statistics and to train and test the DNNs is made available at https://github.com/HOPLAB-LBP/animacy-perception^85^. A minimal dataset to reproduce the results can be found at https://osf.io/7xcg9/.

## References

[1] Kanwisher, N., McDermott, J. & Chun, M. M. The fusiform face area: a module in human extrastriate cortex specialized for face perception. *J. Neurosci.* **17**, 4302–4311 (1997). 9151747 10.1523/JNEUROSCI.17-11-04302.1997 PMC6573547

[2] Tsao, D. Y., Freiwald, W. A., Tootell, R. B. H. & Livingstone, M. S. A cortical region consisting entirely of face-selective cells. *Science* **311**, 670–675 (2006). 16456083 10.1126/science.1119983 PMC2678572

[3] Downing, P. E., Jiang, Y., Shuman, M. & Kanwisher, N. A cortical area selective for visual processing of the human body. *Science* **293**, 2470–2473 (2001). 11577239 10.1126/science.1063414

[4] Peelen, M. V. & Downing, P. E. Category selectivity in human visual cortex: Beyond visual object recognition. *Neuropsychologia* **105**, 177–183 (2017). 28377161 10.1016/j.neuropsychologia.2017.03.033

[5] Caramazza, A. & Shelton, J. R. Domain-specific knowledge systems in the brain: the animate-inanimate distinction. *J. Cogn. Neurosci.* **10**, 1–34 (1998). 9526080 10.1162/089892998563752

[6] Cichy, R. M., Pantazis, D. & Oliva, A. Resolving human object recognition in space and time. *Nat. Neurosci.* **17**, 455–462 (2014). 24464044 10.1038/nn.3635 PMC4261693

[7] Rogers, T. T. et al. Evidence for a deep, distributed and dynamic code for animacy in human ventral anterior temporal cortex. *eLife* **10**, 1–32 (2021). 10.7554/eLife.66276 PMC8550752 34704935

[8] Sha, L. et al. The animacy continuum in the human ventral vision pathway. *J. Cogn. Neurosci.* **27**, 665–678 (2015). 25269114 10.1162/jocn_a_00733

[9] Thorat, S., Proklova, D. & Peelen, M. V. The nature of the animacy organization in human ventral temporal cortex. *eLife* **8**, 1–18 (2019). 10.7554/eLife.47142 PMC6733573 31496518

[10] Ritchie, J. B. et al. Untangling the animacy organization of occipitotemporal cortex. *J. Neurosci.* **41**, 7103–7119 (2021). 34230104 10.1523/JNEUROSCI.2628-20.2021 PMC8372013

[11] Proklova, D., Kaiser, D. & Peelen, M. V. Disentangling representations of object shape and object category in human visual cortex: the animate-inanimate distinction. *J. Cogn. Neurosci.* **28**, 680–692 (2016). 26765944 10.1162/jocn_a_00924

[12] Konkle, T. & Caramazza, A. Tripartite organization of the ventral stream by animacy and object size. *J. Neurosci.* **33**, 10235–10242 (2013). 23785139 10.1523/JNEUROSCI.0983-13.2013 PMC3755177

[13] Bracci, S., Ritchie, J. B., Kalfas, I. & Op de Beeck, H. P. The ventral visual pathway represents animal appearance over animacy, unlike human behavior and deep neural networks. *J. Neurosci.* **39**, 6513–6525 (2019). 31196934 10.1523/JNEUROSCI.1714-18.2019 PMC6697402

[14] Contini, E. W., Goddard, E., Grootswagers, T., Williams, M. & Carlson, T. A humanness dimension to visual object coding in the brain. *NeuroImage* **221**, 117139 (2020). 32663643 10.1016/j.neuroimage.2020.117139

[15] He, C., Hung, S. C. & Cheung, O. S. Roles of category, shape, and spatial frequency in shaping animal and tool selectivity in the occipitotemporal cortex. *J. Neurosci.* **40**, 5644–5657 (2020). 32527983 10.1523/JNEUROSCI.3064-19.2020 PMC7363473

[16] Heider, F. & Simmel, M. An experimental study of apparent behavior. *Am. J. Psychol.* **57**, 243–259 (1944).

[17] Michotte, A. *The Perception of Causality* (Oxford: Basic Books, 1963).

[18] Scholl, B. J. & Tremoulet, P. D. Perceptual causality and animacy. *Trends Cogn. Sci.* **4**, 299–309 (2000). 10904254 10.1016/s1364-6613(00)01506-0

[19] Tremoulet, P. D. & Feldman, J. Perception of animacy from the motion of a single object. *Perception* **29**, 943–951 (2000). 11145086 10.1068/p3101

[20] Kazanas, S. A., Altarriba, J. & O’Brien, E. G. Paired-associate learning, animacy, and imageability effects in the survival advantage. *Mem. Cognition* **48**, 244–255 (2020). 10.3758/s13421-019-01007-2 31916198

[21] Nairne, J. S., Thompson, S. R. & Pandeirada, J. N. Adaptive memory: survival processing enhances retention. *J. Exp. Psychol.: Learn. Mem. Cognition* **33**, 263–273 (2007). 10.1037/0278-7393.33.2.263 17352610

[22] Wardle, S. G., Taubert, J., Teichmann, L. & Baker, C. I. Rapid and dynamic processing of face pareidolia in the human brain. *Nat. Commun.* **11**, 1–14 (2020). 32908146 10.1038/s41467-020-18325-8 PMC7481186

[23] Dobs, K., Martinez, J., Kell, A. J. E. & Kanwisher, N. Brain-like functional specialization emerges spontaneously in deep neural networks. *Sci. Adv.* **8**, eabl8913 (2022). 35294241 10.1126/sciadv.abl8913 PMC8926347

[24] Jacob, G., Pramod, R. T., Katti, H. & Arun, S. P. Qualitative similarities and differences in visual object representations between brains and deep networks. *Nat. Commun.* **12**, 1–14 (2021). 33767141 10.1038/s41467-021-22078-3 PMC7994307

[25] Khaligh-Razavi, S.-M. & Kriegeskorte, N. Deep supervised, but not unsupervised, models may explain IT cortical representation. *PLoS Comput. Biol.* **10**, e1003915 (2014). 25375136 10.1371/journal.pcbi.1003915 PMC4222664

[26] King, M. L., Groen, I. I., Steel, A., Kravitz, D. J. & Baker, C. I. Similarity judgments and cortical visual responses reflect different properties of object and scene categories in naturalistic images. *NeuroImage* **197**, 368–382 (2019). 31054350 10.1016/j.neuroimage.2019.04.079 PMC6591094

[27] Kubilius, J., Bracci, S. & Op de Beeck, H. P. Deep neural networks as a computational model for human shape sensitivity. *PLoS Comput. Biol.* **12**, 1–26 (2016). 10.1371/journal.pcbi.1004896 PMC4849740 27124699

[28] Zeman, A. A., Ritchie, J. B., Bracci, S. & Op de Beeck, H. Orthogonal representations of object shape and category in deep convolutional neural networks and human visual cortex. *Sci. Rep.* **10**, 1–12 (2020). 32051467 10.1038/s41598-020-59175-0 PMC7016009

[29] Güçlü, U. & van Gerven, M. A. Deep neural networks reveal a gradient in the complexity of neural representations across the ventral stream. *J. Neurosci.* **35**, 10005–10014 (2015). 26157000 10.1523/JNEUROSCI.5023-14.2015 PMC6605414

[30] Eickenberg, M., Gramfort, A., Varoquaux, G. & Thirion, B. Seeing it all: convolutional network layers map the function of the human visual system. *NeuroImage* **152**, 184–194 (2017). 27777172 10.1016/j.neuroimage.2016.10.001

[31] Cichy, R. M., Khosla, A., Pantazis, D., Torralba, A. & Oliva, A. Comparison of deep neural networks to spatio-temporal cortical dynamics of human visual object recognition reveals hierarchical correspondence. *Sci. Rep.* **6**, 1–13 (2016). 27282108 10.1038/srep27755 PMC4901271

[32] Rajalingham, R. et al. Large-scale, high-resolution comparison of the core visual object recognition behavior of humans, monkeys, and state-of-the-art deep artificial neural networks. *J. Neurosci.* **38**, 7255–7269 (2018). 30006365 10.1523/JNEUROSCI.0388-18.2018 PMC6096043

[33] Linsley, D., Eberhardt, S., Sharma, T., Gupta, P. & Serre, T. What are the visual features underlying human versus machine vision? In *Proc. IEEE International Conference on Computer Vision Workshops* 2706–2714 (IEEE, 2017).

[34] Baker, N. & Elder, J. H. Deep learning models fail to capture the configural nature of human shape perception. *Science* **25** (2022). 10.1016/j.isci.2022.104913 PMC9429800 36060067

[35] Bracci, S. & Op De Beeck, H. P. Understanding human object vision: a picture is worth a thousand representations. *Annu. Rev. Psychol.* **74** (2023). 10.1146/annurev-psych-032720-041031 36378917

[36] Kubilius, J. et al. Brain-like object recognition with high-performing shallow recurrent ANNs. *Adv. Neural Inform. Process. Syst.* **32** (2019).

[37] Kietzmann, T. C. et al. Recurrence is required to capture the representational dynamics of the human visual system. *Proc. Natl Acad. Sci.* **116**, 21854–21863 (2019). 31591217 10.1073/pnas.1905544116 PMC6815174

[38] Yamins, D. L. et al. Performance-optimized hierarchical models predict neural responses in higher visual cortex. *Proc. Natl Acad. Sci.* **111**, 8619–8624 (2014). 24812127 10.1073/pnas.1403112111 PMC4060707

[39] Bowers, J. S. et al. Deep problems with neural network models of human vision. *Behav. Brain Sci.* **46**, e385 (2022). 10.1017/S0140525X22002813 36453586

[40] Achtman, R. L., Hess, R. F. & Wang, Y. Z. Sensitivity for global shape detection. *J. Vis.* **3**, 616–624 (2003). 14640885 10.1167/3.10.4

[41] Simoncelli, E. P. & Olshausen, B. A. Natural image statistics and neural representation. *Annu. Rev. Neurosci.* **24**, 1193–1216 (2001). 11520932 10.1146/annurev.neuro.24.1.1193

[42] Kreiman, G. & Serre, T. Beyond the feedforward sweep: feedback computations in the visual cortex. *Ann. N. Y. Acad. Sci.* **1464**, 222–241 (2020). 32112444 10.1111/nyas.14320 PMC7456511

[43] Angelucci, A. & Bressloff, P. C. Contribution of feedforward, lateral and feedback connections to the classical receptive field center and extra-classical receptive field surround of primate v1 neurons. *Prog. Brain Res.* **154**, 93–120 (2006). 17010705 10.1016/S0079-6123(06)54005-1

[44] Jia, K. et al. Recurrent processing drives perceptual plasticity. *Curr. Biol.* **30**, 4177–4187 (2020). 32888488 10.1016/j.cub.2020.08.016 PMC7658806

[45] Lamme, V. A. F. & Roelfsema, P. R. The distinct modes of vision offered by feedforward and recurrent processing. *Trends Neurosci.* **23**, 571–579 (2000). Publisher: Elsevier. 11074267 10.1016/s0166-2236(00)01657-x

[46] O’Reilly, R. C., Wyatte, D., Herd, S., Mingus, B. & Jilk, D. J. Recurrent processing during object recognition. *Front. Psychol.* **4**, 124 (2013). 23554596 10.3389/fpsyg.2013.00124 PMC3612699

[47] Palmeri, T. J., Wong, A. C. N. & Gauthier, I. Computational approaches to the development of perceptual expertise. *Trends Cogn. Sci.* **8**, 378–386 (2004). 15335465 10.1016/j.tics.2004.06.001

[48] Charest, I., Allen, E., Wu, Y., Naselaris, T. & Kay, K. Precise identification of semantic representations in the human brain. *J. Vis.* **20**, 539 (2020).

[49] Zhuang, C., Wang, Y., Yamins, D. & Hu, X. Deep learning predicts correlation between a functional signature of higher visual areas and sparse firing of neurons. *Front. Comput. Neurosci.* **11** (2017). 10.3389/fncom.2017.00100 PMC5670118 29163117

[50] Ahn, S., Zelinsky, G. J. & Lupyan, G. Use of superordinate labels yields more robust and human-like visual representations in convolutional neural networks. *J. Vis.* **21**, 1–19 (2021). 10.1167/jov.21.13.13 PMC8727315 34967860

[51] Geirhos, R. et al. ImageNet-trained CNNs are biased towards texture; increasing shape bias improves accuracy and robustness. Preprint at https://arxiv.org/abs/1811.12231 (2019).

[52] Mehrer, J., Spoerer, C. J., Jones, E. C., Kriegeskorte, N. & Kietzmann, T. C. An ecologically motivated image dataset for deep learning yields better models of human vision. *Proc. Natl Acad. Sci. USA* **118**, 1–9 (2021). 10.1073/pnas.2011417118 PMC7923360 33593900

[53] Friston, K. A theory of cortical responses. *Philos. Trans. R. Soc. B: Biol. Sci.* **360**, 815–836 (2005). 10.1098/rstb.2005.1622 PMC1569488 15937014

[54] Huang, Y. & Rao, R. P. N. Predictive coding. *Wiley Interdiscip. Rev.: Cogn. Sci.* **2**, 580–593 (2011). 26302308 10.1002/wcs.142

[55] Rao, R. P. N. & Ballard, D. H. Predictive coding in the visual cortex: a functional interpretation of some extra-classical receptive-field effects. *Nat. Neurosci.* **2**, 79–87 (1999). 10195184 10.1038/4580

[56] Pang, Z., O’May, C. B., Choksi, B. & VanRullen, R. Predictive coding feedback results in perceived illusory contours in a recurrent neural network. *Neural Netw.* **144**, 164–175 (2021). 34500255 10.1016/j.neunet.2021.08.024

[57] Choksi, B. et al. Predify: Augmenting deep neural networks with brain-inspired predictive coding dynamics. *Adv. Neural Inf. Process. Syst.* **34**, 14069–14083 (2021).

[58] Jozwik, K. M., Kietzmann, T. C., Cichy, R. M., Kriegeskorte, N. & Mur, M. Deep neural networks and visuo-semantic models explain complementary components of human ventral-stream representational dynamics. *J. Neurosci.* **43**, 1731–1741 (2023). 36759190 10.1523/JNEUROSCI.1424-22.2022 PMC10010451

[59] Doerig, A. et al. Semantic scene descriptions as an objective of human vision. Preprint at https://arxiv.org/abs/2209.11737 (2022).

[60] Wang, A. Y., Kay, K., Naselaris, T., Tarr, M. J. & Wehbe, L. Incorporating natural language into vision models improves prediction and understanding of higher visual cortex. *BioRxiv*10.1101/2022.09.27.508760 (2022).

[61] Mokady, R., Hertz, A. & Bermano, A. H. Clipcap: Clip prefix for image captioning. Preprint at https://arxiv.org/abs/2111.09734 (2021).

[62] Krizhevsky, A., Sutskever, I. & Hinton, G. E. Imagenet classification with deep convolutional neural networks. *Adv. Neural Inform. Process. Syst.* **25** (2012).

[63] Storrs, K. R., Kietzmann, T. C., Walther, A., Mehrer, J. & Kriegeskorte, N. Diverse deep neural networks all predict human inferior temporal cortex well, after training and fitting. *J. Cogn. Neurosci.* **33**, 2044–2064 (2021). 34272948 10.1162/jocn_a_01755

[64] Chen, C.-Y., Leys, G., Bracci, S. & Op de Beeck, H. The representational dynamics of the animal appearance bias in human visual cortex are indicative of fast feedforward processing. *Imaging Neurosci.* **1**, 1–26 (2023). 10.1162/imag_a_00006 PMC12007534 40799706

[65] Dobs, K., Yuan, J., Martinez, J. & Kanwisher, N. Behavioral signatures of face perception emerge in deep neural networks optimized for face recognition. *Proc. Natl Acad. Sci.* **120**, e2220642120 (2023). 37523537 10.1073/pnas.2220642120 PMC10410721

[66] Feichtenhofer, C., Fan, H., Malik, J. & He, K. Slowfast networks for video recognition. In *Proc. IEEE/CVF International Conference on Computer Vision* 6202–6211 (IEEE, 2019).

[67] Konkle, T. & Alvarez, G. A. A self-supervised domain-general learning framework for human ventral stream representation. *Nat. Commun.* **13** (2022). 10.1038/s41467-022-28091-4 PMC8789817 35078981

[68] Spriet, C., Abassi, E., Hochmann, J.-R. & Papeo, L. Visual object categorization in infancy. *Proc. Natl Acad. Sci.* **119**, e2105866119 (2022). Publisher: National Acad Sciences. 35169072 10.1073/pnas.2105866119 PMC8872728

[69] Kiani, R., Esteky, H., Mirpour, K. & Tanaka, K. Object category structure in response patterns of neuronal population in monkey inferior temporal cortex. *J. Neurophysiol.* **97**, 4296–4309 (2007). 17428910 10.1152/jn.00024.2007

[70] Arcaro, M. J. & Livingstone, M. S. On the relationship between maps and domains in inferotemporal cortex. *Nat. Rev. Neurosci.* **22**, 573–583 (2021). 34345018 10.1038/s41583-021-00490-4 PMC8865285

[71] Jozwik, K. M., Kriegeskorte, N., Storrs, K. R. & Mur, M. Deep convolutional neural networks outperform feature-based but not categorical models in explaining object similarity judgments. *Front. Psychol.* **8**, 1726 (2017). 29062291 10.3389/fpsyg.2017.01726 PMC5640771

[72] Kriegeskorte, N. Deep neural networks: a new framework for modeling biological vision and brain information processing. *Annu. Rev. Vis. Sci.* **1**, 417–446 (2015). 28532370 10.1146/annurev-vision-082114-035447

[73] Lindsay, G. Convolutional neural networks as a model of the visual system: past, present, and future. *J. Cogn. Neurosci.* **33**, 2017–2031 (2020). 10.1162/jocn_a_01544 32027584

[74] Kriegeskorte, N. & Douglas, P. K. Cognitive computational neuroscience. *Nat. Neurosci.* **21**, 1148–1160 (2018). 30127428 10.1038/s41593-018-0210-5 PMC6706072

[75] Griffin, G., Holub, A. & Perona, P. *Caltech-256 Object Category Dataset.* Technical Report 7694 (California Institute of Technology, 2007).

[76] Choi, Y., Uh, Y., Yoo, J. & Ha, J. W. StarGAN v2: diverse image synthesis for multiple domains. In *Proc. IEEE Computer Society Conference on Computer Vision and Pattern Recognition* 8185–8194 (IEEE Computer Society, 2020).

[77] Kang, D. W. et al. Structural and functional connectivity changes beyond visual cortex in a later phase of visual perceptual learning. *Sci. Rep.* **8**, 1–9 (2018). 29581455 10.1038/s41598-018-23487-z PMC5979999

[78] Lee, D. Which deep learning model can best explain object representations of within-category exemplars? *J. Vis.* **21**, 12 (2021). 34520508 10.1167/jov.21.10.12 PMC8444465

[79] Wenliang, L. & Seitz, A. R. Deep neural networks for modeling visual perceptual learning. *J. Neurosci.* **38**, 1620–17 (2018). 10.1523/JNEUROSCI.1620-17.2018 PMC6031581 29793979

[80] Zulkeflie, S. A., Fammy, F. A., Ibrahim, Z. & Sabri, N. Evaluation of basic convolutional neural network, alexnet and bag of features for indoor object recognition. *Int. J. Mach. Learn. Comput.* **9**, 801–806 (2019).

[81] Alamia, A., Mozafari, M., Choksi, B. & VanRullen, R. On the role of feedback in visual processing: a predictive coding perspective. Preprint at https://arxiv.org/abs/2106.04225 (2021). 10.1016/j.neunet.2022.10.020 36375346

[82] Radford, A. et al. Learning transferable visual models from natural language supervision. In: *Proc. 38th International Conference on Machine Learning*, vol. 139 of *Proceedings of Machine Learning Research* (eds. Meila, M. & Zhang, T.) 8748–8763 (PMLR, 2021). https://proceedings.mlr.press/v139/radford21a.html.

[83] Elkin, L. A., Kay, M., Higgins, J. J. & Wobbrock, J. O. An aligned rank transform procedure for multifactor contrast tests. In *The 34th Annual ACM Symposium on User Interface Software and Technology* 754–768 (ACM, 2021).

[84] Grill-Spector, K. & Weiner, K. S. The functional architecture of the ventral temporal cortex and its role in categorization. *Nat. Rev. Neurosci.* **15**, 536–548 (2014). 24962370 10.1038/nrn3747 PMC4143420

[85] SDuyck & Costantino, A. HOPLAB-LBP/animacy-perception: CommsBio submission DOI (2024). 10.5281/zenodo.14165547.

