# Supplementary material for: A computational deep learning investigation of animacy perception in the human brain
Source: Commun Biol. 2024 Dec 31;7:1718. doi: 10.1038/s42003-024-07415-8 (PMC11688457; doi:10.1038/s42003-024-07415-8)
Supplement: Supplementary file 1 — SUPPLEMENTAL MATERIAL [file 42003_2024_7415_MOESM1_ESM.pdf]

1. Alternative network and extraction layer

This section provides a summary of our findings following a similar analysis to the one reported in the main text for an alternative network (VGG, Supplementary Figure 1) and an alternative extraction layer (FC7 of Alexnet, Supplementary Figures 2).

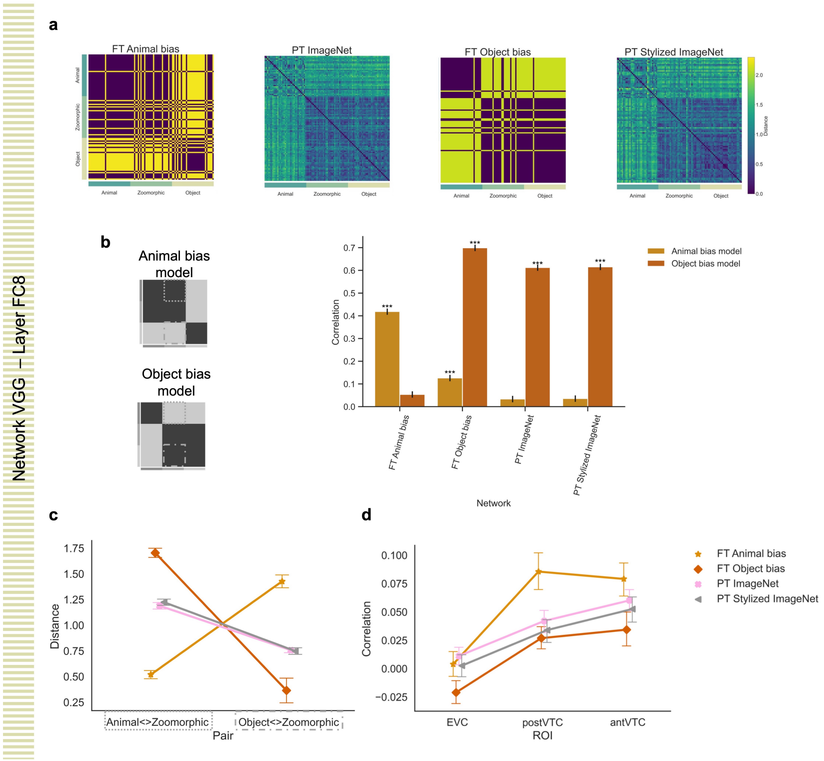

Supplementary Figure 1: **Overview of main findings in VGG layer FC8.** **(a)** The Representational Dissimilarity Matrices of four networks included in the analyses are displayed. PT = pretrained VGG, FT = fine-tuned VGG. **(b)** Graphical display of the independent Animal bias model and Object bias model. **(c)** The graph represents the correlation for each neural network with each bias model. Significant values (i.e., *** $p < .0001$, ** $p < .001$, * $p < .01$), were computed with permutation tests (10,000 randomizations), and error bars indicate the standard error calculated by bootstrapping. **(d)** Mean distance score of each image in the contrast Animal<> Zoomorphic and Object <> Zoomorphic and this for each neural network, showing the same effect as for AlexNet so that only for FT Animal Bias we see a strongly upward curve (Animal<> zoomorphic smaller than Object <>Zoomorphic). e Graph with the individual correlations between the neural data in the three regions of interest (i.e., EVC, posterior- and anterior VTC) and the neural network data resulting from the different training regimes. For VGG we also found, as before for AlexNet, that FT Animal Bias has the strongest correlation with the neural data, although in case of VGG this effect is most prominent for posterior VTC. All error bars represent the standard error. VGG-19 is used for all neural networks, except for PT Stylized ImageNet, which was available in VGG-16.

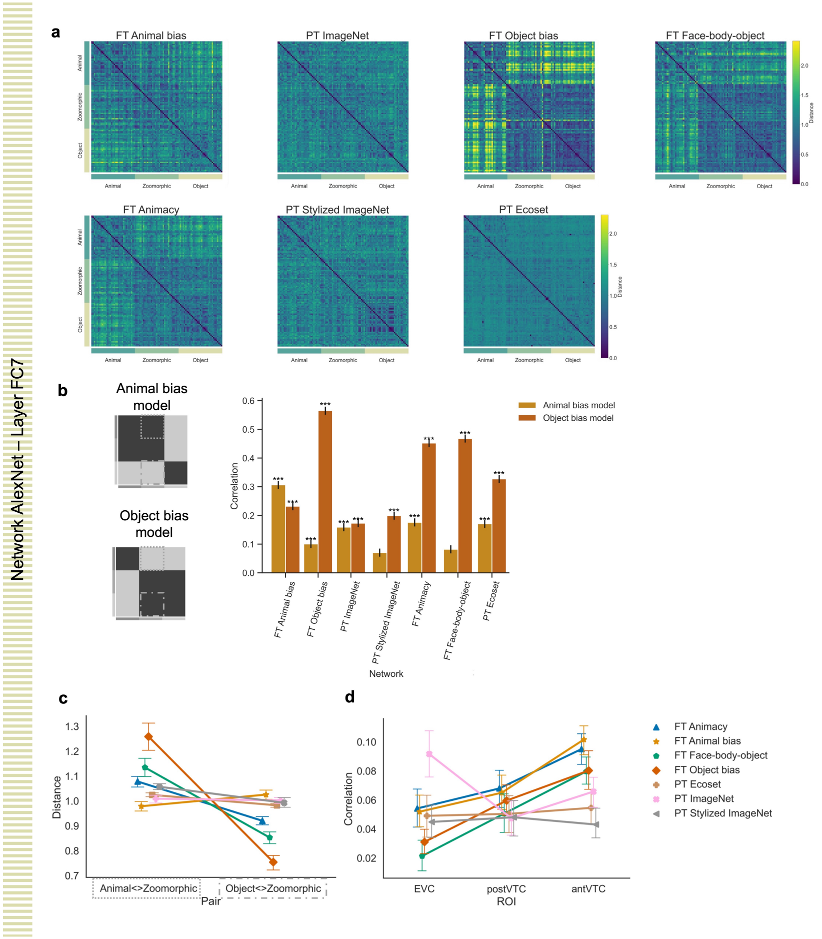

Supplementary Figure 2: **Overview of main and alternative findings in AlexNet layer FC7.** **(a)** The Representational Dissimilarity Matrices of three main networks included in the analyses are displayed. PT = pretrained AlexNet, FT = fine-tuned AlexNet. **(b)** Graphical display of the independent Animal bias model and Object bias model. **(c)** The graph represents the correlation for all tested neural networks with each bias model. Significant values (i.e., *** $p < .0001$, ** $p < .001$, * $p < .01$), were computed with permutation tests (10,000 randomizations), and error bars indicate standard error computed via bootstrapping. **(d)** Mean distance score of each image in the contrast Animal <> Zoomorphic and Object <> Zoomorphic and this for each neural network. Also in FC7 we find that FT Animal Bias tends to have the most upward going curve (Animal <> zoomorphic smaller than Object <> Zoomorphic), but the difference with the other networks is much smaller than for FC8. **(e)** Graph with the individual correlations between the neural data in the three regions of interest (i.e., EVC, posterior- and anterior VTC) and the neural network data resulting from the different training regimes. Also in FC7 the correlation with VTC representations tends to be the highest for FT Animal Bias, but again the effects are small compared to findings in FC8. The relative effect size in different layers might depend upon meta-parameters such as learning rate and how many layers are fixed during fine-tuning, yet it is reassuring that effects go in similar directions in different layers. All error bars represent the standard error.

2. Additional training conditions

To further explore the potential for inducing an animal bias in DNN representations, we performed additional analyses using three alternative network architectures and training paradigms: an AlexNet trained on face recognition [1], a Video ResNet [2] pretrained on action recognition using the Kinetics dataset, and an AlexNet trained with a contrastive learning algorithm [3]. These analyses aimed to investigate whether alternative constraints or learning approaches could produce an animal bias in the network representations. We constructed RDMs from the activations of the final fully connected layer (FC8 or equivalent) for each of these networks and correlated them with our independent model RDMs (i.e., Animal bias, Object bias) (Figure 3).

Our findings largely aligned with the main results reported in the text. The AlexNet trained with contrastive learning exhibited a stronger correlation with the object bias model ($r = .14$, $p < .001$) compared to the animal bias model ($r = .12$, $p < .001$). The AlexNet trained on face recognition showed a weak negative correlation with the object bias model ($r = -.06$, $p = .003$) and a weak positive correlation with the animal bias model ($r = .13$, $p < .001$). It is important to note that while a significant positive correlation exists between the animal bias model and the face-trained AlexNet, the strength of this correlation is substantially weaker than that observed between the same independent model and the RDM of the FT Animal bias DNN reported in the main text ($r = .57$). The Video ResNet trained on action recognition showed very weak correlations with both bias models (animal bias: $r = .01$, $p = .728$; object bias: $r = -.02$, $p = .421$), neither reaching statistical significance.

These results corroborate our main findings, demonstrating that even with alternative training regimes or learning algorithms, DNNs do not consistently develop a strong animal bias in their representational geometry. This further supports the robustness of our primary conclusions regarding the nature of animal bias in DNN representations.

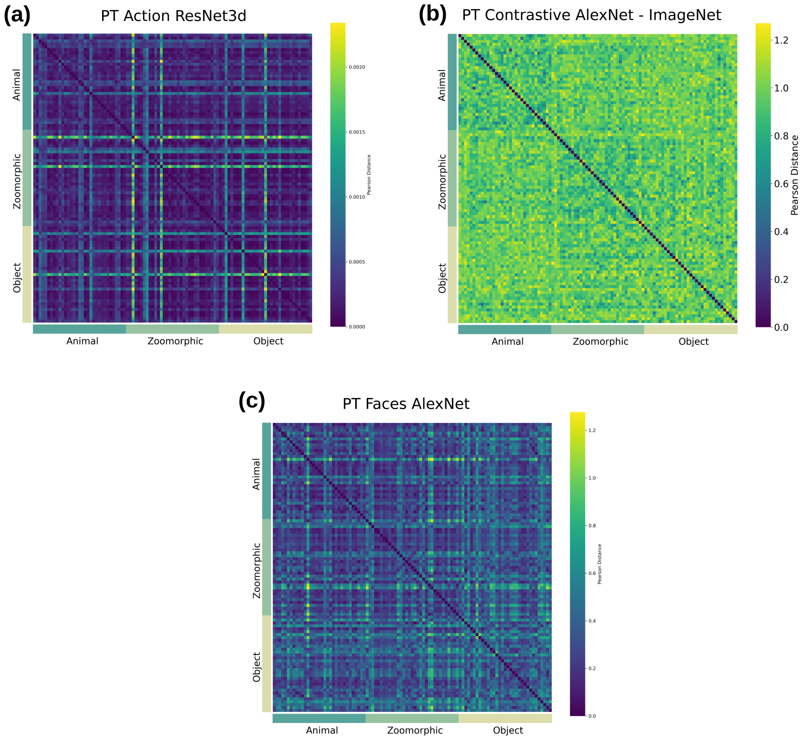

Supplementary Figure 3: **Representational dissimilarity matrices of alternative training regimes.** Representational Dissimilarity Matrices (RDMs) for three alternative network architectures and training regimes: (a) AlexNet trained with contrastive learning, (b) AlexNet trained on face recognition, and (c) Video ResNet trained on action recognition. Each RDM shows the pairwise dissimilarities between stimulus representations in the final layer of the respective network. Warmer colors indicate higher dissimilarity. These visualizations complement the correlation analyses described in the text, illustrating the representational structure learned by each network under different training regimes.

3. Distance scores distributions

Figure 4 presents violin plots of the distance scores for the Animal<>Zoomorphic and Object<>Zoomorphic contrasts across all DNNs reported in the main text in the FC8 layer (or equivalent).

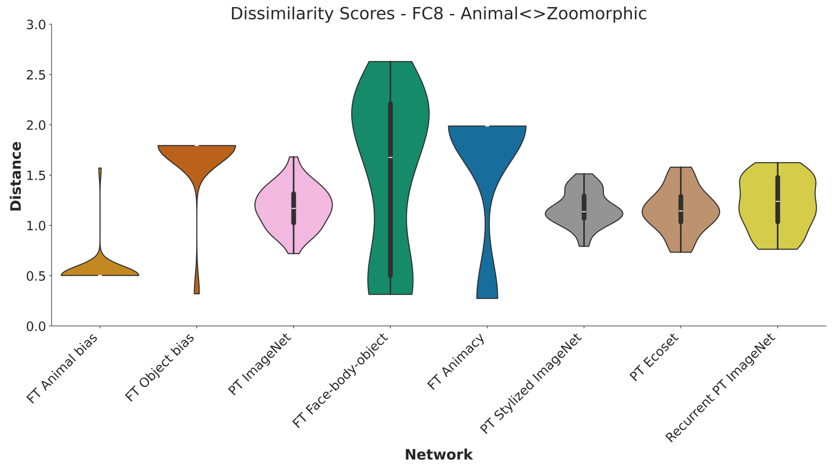

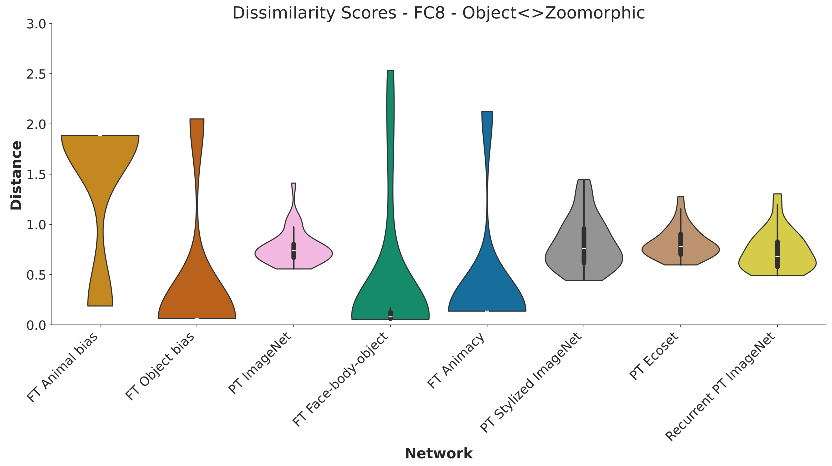

Supplementary Figure 4: **Comparison of distance scores across conditions in FC8 layer.** Violin plots showing the distance scores between images in the Animal<>Zoomorphic (top) and Object<>Zoomorphic (bottom) contrasts across all tested neural networks in the FC8 layer. The y-axis represents the dissimilarity value, while the x-axis corresponds to a different condition as detailed in the main text.

4. Bayes Factor Supplementary Information

Supplementary Figure 5 presents the Bayes Factors (BF_{10}) for pairwise distance comparisons between models for the Animal<>Zoomorphic and Object<>Zoomorphic contrasts in the FC8 layer (or equivalent). Subplots (a) and (b) display the original Bayes Factors for Animal<>Zoomorphic and Object<>Zoomorphic comparisons, respectively. Subplots (c) and (d) present the same data, but with BF values transformed to log10 scale for enhanced visualization and interpretation.

The Bayes Factor (BF_{10}) quantifies evidence for the alternative hypothesis (H_1) relative to the null hypothesis (H_0), indicating the likelihood of differences versus similarities in distances between models. $\mathrm{BF}_{10} > 100$ ($\log_{10}(\mathrm{BF}_{10}) > 2$) suggests extreme evidence for H_1, while $\mathrm{BF}_{10} < 1/100$ ($\log_{10}(\mathrm{BF}_{10}) <$ -2) indicates extreme evidence for H_0. When $\mathrm{BF}_{10} = 1$ ($\log_{10}(\mathrm{BF}_{10}) = 0$), there is no or poor evidence favoring either hypothesis. Between these extremes, $\mathrm{BF}_{10} > 1$ supports H_1 and $\mathrm{BF}_{10} < 1$ supports H_0, with larger absolute values of $\log_{10}(\mathrm{BF}_{10})$ indicating stronger evidence.

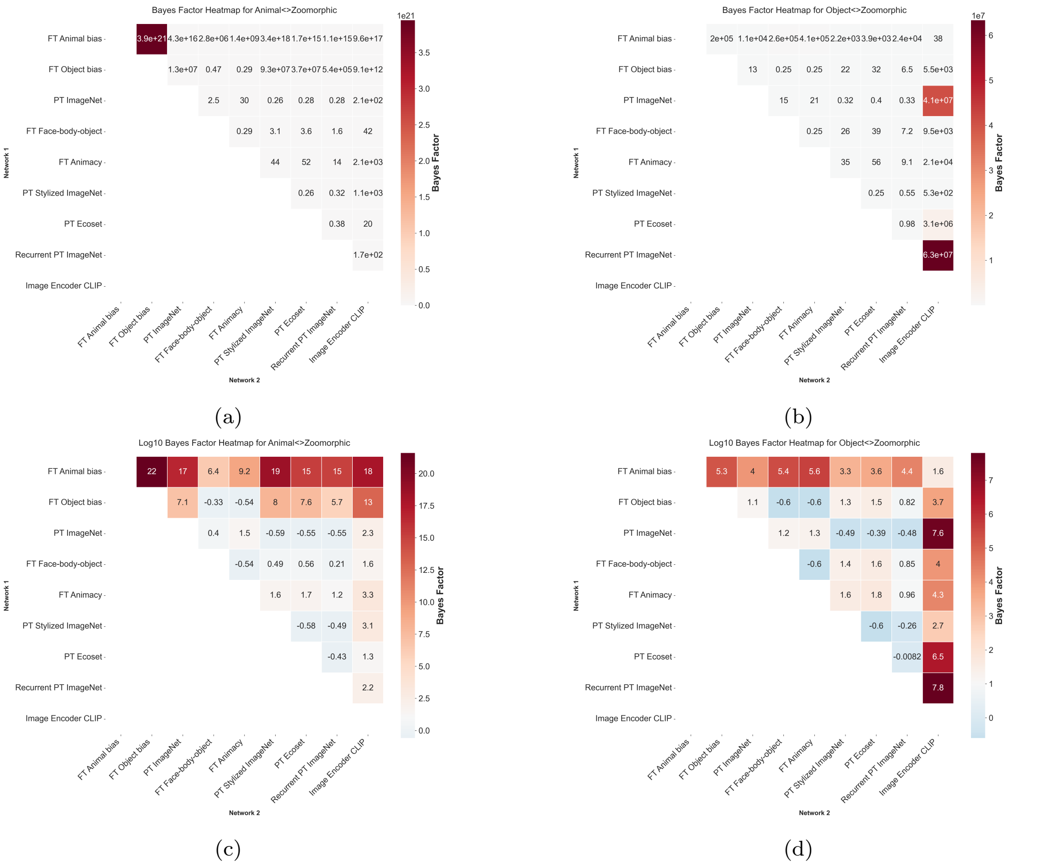

Supplementary Figure 5: **Bayes Factors (BF) for pairwise model comparisons**. (a) Original BF10 for Animal<>Zoomorphic contrasts. (b) Original BF10 for Object<>Zoomorphic contrasts. (c) Log10-transformed BF10 for Animal<>Zoomorphic contrasts. (d) Log10-transformed BF10 for Object<>Zoomorphic contrasts. The color scale indicates the strength of evidence for similarity or difference between model distances, with warmer colors representing stronger evidence for differences and cooler colors representing stronger evidence for similarities.

References

[1] K. Dobs, J. Yuan, J. Martinez, N. Kanwisher, Behavioral signatures of face perception emerge in deep neural networks optimized for face recognition, Proceedings of the National Academy of Sciences 120 (32) (2023) e2220642120.

[2] C. Feichtenhofer, H. Fan, J. Malik, K. He, Slowfast networks for video recognition, in: Proceedings of the IEEE/CVF international conference on computer vision, 2019, pp. 6202–6211.

[3] T. Konkle, G. A. Alvarez, Beyond category-supervision: Computational support for domain-general pressures guiding human visual system representation, bioRxiv (2021).
